# Comparative Characterization of *Aspergillus* Pectin Lyases by Discriminative Substrate Degradation Profiling

**DOI:** 10.3389/fbioe.2020.00873

**Published:** 2020-07-30

**Authors:** Birgitte Zeuner, Thore Bach Thomsen, Mary Ann Stringer, Kristian B. R. M. Krogh, Anne S. Meyer, Jesper Holck

**Affiliations:** ^1^Protein Chemistry and Enzyme Technology, Department of Biotechnology and Biomedicine, Technical University of Denmark, Lyngby, Denmark; ^2^Novozymes A/S, Lyngby, Denmark

**Keywords:** pectin lyase, multigenecity, sugar beet pectin, apple pectin, citrus pectin, product profiling

## Abstract

Fungal genomes often contain several copies of genes that encode carbohydrate active enzymes having similar activity. The copies usually have slight sequence variability, and it has been suggested that the multigenecity represents distinct reaction optima versions of the enzyme. Whether the copies represent differences in substrate attack proficiencies of the enzyme have rarely been considered. The genomes of *Aspergillus* species encode several pectin lyases (EC 4.2.2.10), which all belong to polysaccharide lyase subfamily PL1_4 in the CAZy database. The enzymes differ in terms of sequence identity and phylogeny, and exhibit structural differences near the active site in their homology models. These enzymes catalyze pectin degradation via eliminative cleavage of the α-(1,4) glycosidic linkages in homogalacturonan with a preference for linkages between methyl-esterified galacturonate residues. This study examines four different pectin lyases (PelB, PelC, PelD, and PelF) encoded by the same *Aspergillus* sp. (namely *A. luchuensis*), and further compares two PelA pectin lyases from two related *Aspergillus* spp. (*A. aculeatus* and *A. tubingensis*). We report the phylogeny, enzyme kinetics, and enzymatic degradation profiles of the enzymes’ action on apple pectin, citrus pectin, and sugar beet pectin. All the pectin lyases exerted highest reaction rate on apple pectin [degree of methoxylation (DM) 69%, degree of acetylation (DAc) 2%] and lowest reaction rate on sugar beet pectin (DM 56%, DAc 19%). Activity comparison at pH 5–5.5 produced the following ranking: PelB > PelA > PelD > PelF > PelC. The evolution of homogalacturonan-oligomer product profiles during reaction was analyzed by liquid chromatography with mass spectrometry (LC-MS) detection. This analyses revealed subtle differences in the product profiles indicating distinct substrate degradation preferences amongst the enzymes, notably with regard to acetyl substitutions. The LC-MS product profiling analysis thus disclosed that the multigenecity appears to provide the fungus with additional substrate degradation versatility. This product profiling furthermore represents a novel approach to functionally compare pectin-degrading enzymes, which can help explain structure-function relations and reaction properties of disparate copies of carbohydrate active enzymes. A better understanding of the product profiles generated by pectin modifying enzymes has significant implications for targeted pectin modification in food and biorefinery processes.

## Introduction

**FIGURE 1:**
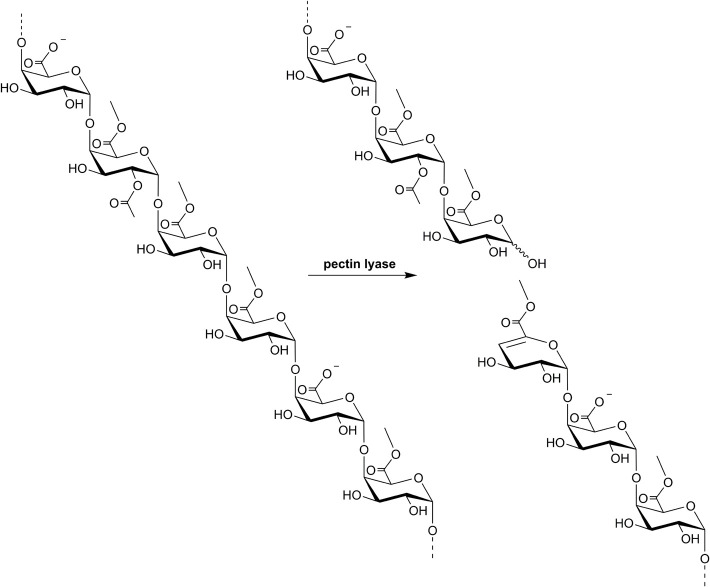
Stylized illustration of a pectin lyase-catalyzed β-elimination reaction on pectin. GalA can be methoxylated on *O*-6 and acetylated on *O*-2 and/or *O*-3. The illustration depicts a pectin fragment substrate with a degree of methoxylation (DM) of 67% and a degree of acetylation (DAc) of 17%, whereas the true substitution degrees vary between pectins of different sources (Table 2). Note the preference for cleavage between methylesterified GalA moieties as outlined in more detail in the text.

Pectin lyases (EC 4.2.2.10) catalyze cleavage of the α-(1,4) glycosidic linkages between methyl-esterified galacturonic acid (GalA) units in the homogalacturonan backbone of pectin through a β-elimination reaction (Figure 1). The β-elimination leads to formation of a 4,5-unsaturated 6-*O*-methylated galacturonide molecule in the non-reducing end of one of the cleavage products, which has absorbance maximum at 235 nm. Pectin lyases thus prefer linkages between methyl-esterified (methoxylated) GalA units, but also catalyze bond cleavage at sites where only the GalA moiety in the +1 subsite is methoxylated, albeit with lower specific activity (Mutenda et al., 2002; Van Alebeek et al., 2002). Accordingly, pectin lyase activity is heavily dependent on the degree of methoxylation (DM) of the substrate, and also on the methyl ester distribution: A blockwise distribution of methoxylated GalA units results in higher pectin lyase activity than a random distribution (Mutenda et al., 2002; Van Alebeek et al., 2002). Detailed studies on *Aspergillus niger* pectin lyase A have further revealed requirement for methoxylated GalA in subsites +1 and +3, whereas subsites +2, +4, and -1 to -4 appear to allow accommodation of unesterified GalA units (Van Alebeek et al., 2002).

Important industrial applications of pectin lyases [usually employed in mixtures with other pectin depolymerizing enzymes, notably endo-polygalacturonases (EC 3.2.1.15)] include wine and juice pre-press maceration as well as juice clarification (Mantovani et al., 2005; Kassara et al., 2019). Uses of enzymes, including pectinase blends and pectin lyases, in food and beverage processing constitute about 30% of the total enzymes market, and a conservative estimate is that the global market value for pectin modifying enzymes is above US$ 100 million and growing. Pectin lyases are also actively investigated for special new uses such as bio-degumming and cleaning of bast fibers (Kohli and Gupta, 2019), and for production of distinct pectic oligosaccharides having prebiotic or anti-inflammatory properties (Chung et al., 2017; Tan et al., 2018). Most commercial pectinases originate from filamentous fungi, notably several derive from the *Aspergillus* section *Nigri* such as *Aspergillus niger* and *A. aculeatus* (Sandri et al., 2011). These fungi are specialized in secreting a broad spectrum of enzymes, including pectin-degrading enzymes, which can degrade and utilize the surrounding biomass (Benoit et al., 2012; de Vries et al., 2017; Kowalczyk et al., 2017).

Genome sequencing of the industrial enzyme-producing strain *A. niger* CBS 513.88 has revealed that this fungus contains multiple genes encoding for the same enzymatic activity (Pel et al., 2007; Andersen et al., 2012). Of the 131 genes encoding secreted polysaccharide-active enzymes, five (3.8%) encode pectin lyases; these five are denominated *pelA*, *pelB*, *pelC*, *pelD*, and *pelF* (Pel et al., 2007). Expression profiling of strains derived from *A. niger* CBS 120.49 has shown that the genes are active at different stages of pectin degradation, suggesting different regulatory mechanisms either related to extent of substrate degradation or to the increased acidification of the medium during pectinase action (de Vries et al., 2002). From studies on closely related *Aspergillus* species, it has furthermore become evident that the different species have different enzymatic strategies for efficient degradation of the same substrate (Benoit et al., 2015; Barrett et al., 2020b). Characterization of the individual gene products can therefore be used to efficiently select enzymes for a specific application, e.g., optimal degradation of a given substrate under a certain set of reaction conditions.

We hypothesized that the multiple copies (multigenecity) of genes encoding for pectin lyases in *Aspergilli* might be related to small differences in substrate attack preferences of the enzymes. Such differences could reflect adaptation to particular substitution patterns on the pectin backbone and materialize as minor variations in the active site region of the enzymes. This study was undertaken to examine PelB, PelC, PelD, and PelF from *A. luchuensis* in terms of substrate specificity, oligomer product profiles, pH-temperature optima, kinetic constants, active site and thermal stability. Two PelA pectin lyases from *A. aculeatus* and *A. tubingensis* were included for comparison. *A. tubingensis* and *A. luchuensis* both belong to the *A. niger* clade (Varga et al., 2011; Hong et al., 2013) and the pectin lyases studied here show > 90% sequence identity to the corresponding enzyme proteins from *A. niger* CBS 513.88 (Table 1). *A. aculeatus* is less closely related, yet all three *Aspergillus spp.* belong to section *Nigri* (Varga et al., 2011; Hong et al., 2013).

**TABLE 1:** Name, origin, and publicly available sequence entries of the *Aspergillus* sp. pectin lyases used in the current work.

Name	Origin^a^	Public sequence	Comparable to *A. niger* CBS 513.88	Seq. id.	MW (kDa)
*Aa*PelA	*A. aculeatus*	–	PelA (A2R3I1)	75%^b^	37.8
*At*PelA	*A. tubingensis*	Swiss-Prot A0A100IK89	PelA (A2R3I1)	98%	37.9
*Al*PelB	*A. luchuensis*	Swiss-Prot G7Y0I4	PelB (A2QFN7)	99%	37.9
*Al*PelC	*A. luchuensis*	Swiss-Prot G7XAF0	PelC (A2QW65)	91%	37.4
*Al*PelD	*A. luchuensis*	Swiss-Prot G7Y107	PelD (A2RBL2)	97%	37.2
*Al*PelF	*A. luchuensis*	Swiss-Prot G7XZ48	PelF (A2R6A1)	99%	47.3

*The amino acid sequence of AaPelA is given in Supplementary Table S3. Comparison and sequence identity of the pectin lyases to the commonly known Aspergillus niger CBS 513.88 pectin lyases (reviewed Swiss-Prot entry IDs indicated) are given. Calculated molecular weight (MW) after signal peptide removal as predicted by SignalP-5.0 server is also indicated. AtPelA was produced recombinantly in A. niger, the rest in A. oryzae. ^a^Swiss-Prot entries G7Y0I4, G7XAF0, G7Y107, and G7XZ48 are listed with Aspergillus kawachii NBRC 4308 as origin. but it has been established that A. kawachii should be named A. luchuensis (Hong et al., 2013) and it is therefore named as such here. Swiss-Prot entry A0A100IK89 is listed as Aspergillus niger in UniProt, but was recently reclassified as A. tubingensis. ^b^BLAST against Swiss-Prot gives highest sequence identity (83–84%) to PelAs from A. fischeri, A. fumigatus, and A. oryzae.*

## Materials and Methods

### Chemicals

Sugar beet pectin was provided by CP Kelco ApS (Lille Skensved, Denmark). Pectin from apple with 70–75% esterification (apple pectin), pectin from citrus peel (citrus pectin), and polygalacturonic acid and all other chemicals were purchased from Sigma-Aldrich (Merck) (Darmstadt, Germany).

### Enzyme Expression and Purification

Genes encoding the full length native pectin lyases (Table 1) were PCR amplified using gene specific primers from either a cDNA pool from *Aspergillus tubingensis* or genomic DNA from *Aspergillus luchuensis* and *Aspergillus aculeatus*. The amplified full-length coding sequences were cloned into *Aspergillus* expression vectors and recombinantly expressed in *Aspergillus niger* for *At*PelA and *Aspergillus oryzae* for the remaining pectin lyases. The enzymes were purified to SDS-PAGE electrophoretic purity (Supplementary Figure S1) using hydrophobic interaction chromatography followed by ion exchange chromatography.

All pectin lyases are classified in the CAZy polysaccharide lyase family 1 (PL1). PL1 comprises pectin lyases (EC 4.2.2.10), pectate lyases (EC 4.2.2.2), and exo-pectate lyases (EC 4.2.2.9). Based on phylogenetic analysis of the catalytic domain, PL1 is currently divided into 13 subfamilies, which to some extent reflects substrate specificity (Lombard et al., 2010). Following CAZy nomenclature, PL1 subfamily X is written PL1_X. Characterized pectin lyases are found in PL1_4 and PL1_8, both of which are monospecific subfamilies comprising pectin lyases only. The six *Aspergillus* pectin lyases studied here all belong to PL1_4.

### *In silico* Studies

The sequences of the six pectin lyases studied in the current work were compared to the reviewed Swiss-Prot entries by BLAST analysis from UniProt. Phylogenetic trees were made in CLC Main Workbench (QIAGEN, Hilden, Germany) using all reviewed Swiss-Prot entries for pectin lyases and the sequences of the pectin lyases studied in the current work. Homology models of the enzymes were generated using SWISS-MODEL (Waterhouse et al., 2018). Conserved amino acids in the substrate binding site were identified and colored using PyMOL (Schrödinger, New York, NY, United States). Homology models and crystal structures were aligned in PyMOL using the *super* function. Multiple sequence alignments were generated with ClustalW in MUSCLE (Madeira et al., 2019). Signal peptides were predicted using the SignalP 5.0 web server (Almagro Armenteros et al., 2019), and the theoretical molecular weight of the secreted enzymes were calculated from the amino acid sequence in CLC Main Workbench. Secondary structure prediction was performed with PSIPRED (Buchan and Jones, 2019). The CUPP webserver for functional annotation was accessed at cupp.info (Barrett and Lange, 2019; Barrett et al., 2020a).

### Substrate Characterization

Apple pectin, citrus pectin, and sugar beet pectin substrates were subjected to acid hydrolysis with 4% sulfuric acid following the NREL method (Sluiter et al., 2012). The resulting monosaccharide composition was determined by HPAEC-PAD using a CarboPac PA1 column (Dionex, Sunnyvale, CA, United States) as described previously (Zeuner et al., 2018).

In order to determine degree of methoxylation (DM) and degree of acetylation (DAc) of the pectin substrates, methyl and acetyl substitutions were released by saponification as described previously (Voragen et al., 1986). The released methanol and acetate were quantified by HPLC (Shimadzu, Kyoto, Japan) essentially as described previously (Agger et al., 2010) using an Aminex HPX-87H ion exclusion column (BioRad, Hercules, CA, United States). Elution was performed over 40 min with 4 mM sulfuric acid at a flow rate of 0.6 mL/min and 63°C. Compounds were detected by refractive index detection using methanol and acetate as external standards. The DM is defined as the percentage of methoxylated GalA moieties out of the total amount of GalA moieties. Similarly, the DAc is defined as the percentage of acetyl substituted GalA units.

### Substrate Screening and Enzyme Activity

The initial reaction rates of the pectin lyases were determined on four different pectin substrates: apple pectin, citrus pectin, sugar beet pectin, and polygalacturonic acid using a substrate concentration of 1 g/L in 100 mM sodium acetate buffer at pH 5. The enzyme dosage was 0.4% (w/w) E/S for *Aa*PelA, *At*PelA, and *Al*PelB, 12.5% (w/w) E/S for *Al*PelC, 0.625% (w/w) E/S for *Al*PelD, and 1.25% (w/w) E/S for *Al*PelF. The pectin lyase activity was monitored at 25°C for 10 min by measuring the change in absorbance at 235 nm using an Infinite 200Pro plate reader (Tecan, Männedorf, Switzerland). This change was converted to product formation using an extinction coefficient of 5500 M^–1^ cm^–1^ for the 4,5-unsaturated galacturonide product (Kester and Visser, 1994). One unit (U) is defined as the amount of enzyme which catalyzes the formation of one μmol of 4,5-unsaturated galacturonide product per min at pH 5.0 and 25°C. In turn, the reported specific activity is given as μmol product released per min per mg enzyme.

### Determination of pH-Temperature Optima and Kinetic Constants

The pH-temperature optima of *Aa*PelA, *At*PelA, *Al*PelB, and *Al*PelD were determined by response surface methodology (RSM) using a two-factor face-centered central composite design of experiments (CCF) with three center points. Each sample was analyzed in duplicate. Statistical analysis, model generation, and graphic illustration was made in JMP 14 Pro (SAS, Cary, NC, United States). All reactions were performed with pectin solutions of 2 g/L where pH was adjusted by NaOH and HCl. For *At*PelA and *Al*PelD a pH range of 3.5–5.5 was employed for all substrates. For *Aa*PelA and *Al*PelB the pH range was 5.5–7.5, except for *Aa*PelA on sugar beet pectin, where it was 4.5–6.5. The tested temperature range was 45–65°C for *Aa*PelA, 50–70°C for *At*PelA, 40–60°C for *Al*PelB, and 40–70°C for *Al*PelD. Enzyme dosages ranged from 0.0125 to 0.05% E/S depending on the initial substrate screening results. Reactions took place in preheated substrate solution for 5 min. The reaction was stopped by heating at 95°C. Finally, the extent of reaction was determined from the difference in absorbance at 235 nm when compared to an enzyme-free solution of the same substrate.

Kinetic constants were determined using the same reaction setup at the pH-temperature optima predicted for each enzyme on each substrate based on the RSM results; for *Aa*PelA on sugar beet pectin where no model was generated, center point values (pH 5.5, 55°C) were used instead. Eight different pectin concentrations in the range from 0.25 to 10 g/L were used. The substrate concentration was converted to molar concentration of bonds between GalA moieties based on the monosaccharide composition (Table 2). Reactions were run in triplicates. Kinetic constants were determined from linear regression in a Hanes-Woolf plot of the data.

**TABLE 2:** Average monosaccharide composition in μmol/g pectin dry matter, degree of acetylation (DAc), and degree of methoxylation (DM) of pectin from apple, citrus, and sugar beet.

Pectin source	Monosaccharide composition (μmol/g pectin dry matter)									DAc	DM
	Fuc	Ara	Rha	Gal	Glc	Xyl	Man	GalA	GlcA		
Apple	6 ± 0.2^a^	157 ± 3^b^	110 ± 1^c^	281 ± 6^c^	237 ± 5^a^	51 ± 1^a^	5 ± 0.3^a^	3875 ± 64^b^	8 ± 1^b^	2 ± 0.1^b^	69 ± 3^a^
Citrus	4 ± 0.2^c^	98 ± 3^c^	152 ± 5^b^	478 ± 16^b^	71 ± 2^b^	15 ± 1^b^	4 ± 0.3^ab^	4195 ± 15^a^	5 ± 1^b^	1 ± 0.1^b^	53 ± 1^b^
Sugar beet	5 ± 0.1^b^	512 ± 11^a^	264 ± 5^a^	532 ± 9^a^	30 ± 1^c^	11 ± 0.5^c^	3 ± 0.4^b^	2940 ± 8^c^	33 ± 1^a^	19 ± 1^a^	56 ± 5^b^

*Standard deviations of triplicates are indicated. Superscript letters indicate significant difference (p < 0.05) across the substrates for each monosaccharide or degree of substitution.*

### Thermal Stability Assessment

A thermal shift assay (TSA) (Lo et al., 2004) was used to determine the melting point (*T*_m_) of the pectin lyases at pH 5. Purified pectin lyase samples were prepared for TSA by diluting to a standard concentration of 0.24 mg/mL in Milli-Q water. SYPRO Orange dye (S6650; Life Technologies, Carlsbad, CA, United States) was diluted 1:200 in assay buffer. The assay buffer was 0.1 M succinic acid, 0.1 M HEPES, 0.1 M CHES, 0.1 M CAPS, 0.15 M KCl, 1 mM CaCl_2_, and 0.01 % Triton X100, adjusted to pH 5. For measurement, 10 μl of diluted enzyme sample was combined with 20 μl of diluted dye in the well of a TSA assay plate (Roche LightCycler 480 Multiwell plate 96, white; Roche, Basel, Switzerland) and the plate was covered with optic sealing foil. Thermal ramping and fluorescence measurements were run in a Roche Lightcycler 480 II machine (Roche, Basel, Switzerland) running with a ramp from 25 to 99°C at a rate of 200°C/h. The data collected was analyzed by Roche LightCycler 480 software (release 1.5.0 SP4). All samples were analyzed in duplicate and averaged.

For *Al*PelF, a different analysis method was used for determining *T*_m_, namely nano-differential scanning fluorimetry (nDSF). The enzyme was diluted to 0.5 mg/mL and buffer exchanged to 50 mM acetic acid (pH 5.0) using a PD SpinTrap G-25 column (GE Healthcare, Uppsala, Sweden). The nDSF experiments were done utilizing a Prometheus NT.48 (NanoTemper, Munich, Germany). The experiments were conducted from 20 to 95°C with a temperature gradient of 200°C/h. The transition temperatures (*T*_m_ values) were obtained from peak values derived from the first-derivative of the signal trace (350/330 nm fluorescence ratio or 330 nm fluorescence) using PR.ThermControl software (NanoTemper, Munich, Germany).

### Product Profiling by LC-ESI-MS and SEC

The pectin degradation product profiles were studied for all six pectin lyases using 10 g/L pectin (apple, citrus, or sugar beet) in acetate buffer at pH 5.5 and 40°C. An equimolar enzyme dosage of 60 nM (corresponding to 0.023% E/S for *At*PelA) was used for all enzymes, but due to low activity enzyme dosages of 600 nM were also included for *Al*PelC and *Al*PelF. After 5 and 20 min, and 2 and 24 h, samples were taken out and the enzymes were inactivated by heating at 95°C for 10 min. The samples were centrifuged (14,000 *g*, 5 min) to remove insoluble particles.

For size exclusion chromatography (SEC) analysis of the pectin degradation profiles, the samples were diluted 4 times in the eluent buffer and filtered (0.22 μm). The SEC analysis was performed by injecting on a TSKgel G3000PW column (300 mm × 7.5 mm) equipped with a TSKgel PWH guard column (7.5 mm × 7.5 mm) (Tosoh Bioscience, Tokyo, Japan) using an UltiMate iso-3100 SD pump and an RI-101 refractive index detector. Elution took place at 40°C using 0.1 M NaNO_3_ with 0.02% NaN_3_ as eluent and a flow rate of 1 mL/min. Pullulan standards (180 Da to 110 kDa) were used as reference.

Identification and relative quantification of pectic oligosaccharides was performed by liquid chromatography electrospray ionization mass spectrometry (LC-ESI-MS) on an Amazon SL iontrap (Bruker Daltonics, Bremen Germany) coupled to an UltiMate 3000 UHPLC from Dionex (Sunnyvale, CA, United States). Samples of 10 μL were injected on a porous graphitized carbon column (Hypercarb PGC, 150 mm × 2.1 mm, 3 μm; Thermo Fisher Scientific, Waltham, MA, United States). The chromatography was performed at 0.4 mL/min at 70 °C on a three-eluent system with eluent A (water), eluent B (acetonitrile), and eluent C (100 mM ammonium acetate pH 5). The elution profile was as follows: 0–1 min, 0% B; 1–30 min, linear gradient to 30% B; 30–35 min, isocratic 30% B; 35–40 min, isocratic 0% B. In addition, a constant level of 10% C was maintained throughout the elution. For every four samples, a cleaning procedure of 0–15 min, isocratic 50% B and C; 15–40 min, isocratic 10% C was performed to avoid build-up of polymeric pectin. The electrospray was operated in negative or positive mode with UltraScan mode and a scan range from 100 to 2000 *m*/*z*, smart parameter setting of 500 *m*/*z*, capillary voltage at 4.5 kV, end plate off-set 0.5 kV, nebulizer pressure at 3.0 bar, dry gas flow at 12.0 L/min, and dry gas temperature at 280°C. Identification of observed compounds by *m*/*z* and MS^2^ fragmentation pattern was performed in DataAnalysis 4.2 and relative quantification based on intensity peaks in positive mode was performed in Compass QuantAnalysis 2.2 (Bruker Daltonics, Bremen Germany).

All samples were investigated with respect to their content of all confirmed and putative compounds. The intensity of each compound was defined as the area under the curve of a given extracted ion chromatogram. In order to investigate the product profile, or fingerprint, of each enzyme rather than their overall activity/productivity, all intensities were normalized with respect to the most abundant compound in a given enzyme-substrate-time combination. Data was clustered by hierarchical clustering with complete linkage on the euclidian distance matrix and visualized in the pheatmap-package using R version 3.6.0.

A representative sample (*At*PelA, sugar beet pectin, 24 h) was selected for validation of the method. Three consecutive injections were made for technical replicates. The average coefficient of variation (CV) of the normalized values for all compounds was 9.0% (±5.5%) with no observed linearity between CV% and compound average (data not shown).

### Statistics

One-way ANOVA for determination of statistical significance was performed with JMP 14 Pro (SAS, Cary, NC, United States). Statistical significance was established at *p* < 0.05.

## Results and Discussion

### Pectin Lyase Identity, Phylogeny, and Homology Modeling

The multigenecity of pectinolytic enzymes from *Aspergilli* has been the subject of several studies over the last three decades (Harmsen et al., 1990; Kusters-van Someren et al., 1992; de Vries et al., 2002; Martens-Uzunova and Schaap, 2009; Benoit et al., 2012; Kowalczyk et al., 2017; He et al., 2018). Differences in pH optima (Supplementary Table S1) and expression inducers may partially explain the large gene families observed for several different classes of pectinolytic enzymes (Martens-Uzunova and Schaap, 2009; Andersen et al., 2012), but there is a need for rigorous biochemical characterization of the gene products including specificity toward pectins of different origin and in particular determination of product profiles in order to fully understand this multigenecity. In fact, several cases exist where one of the genes in a pectinolytic gene family is present in one *Aspergillus niger* strain, but absent from another (Martens-Uzunova and Schaap, 2009). For instance, the genome of *A. nig*er ATCC 1015 harbors a putatively pectin lyase-encoding *pelE* gene, but there is no evidence of such a gene in the genome of *A. niger* CBS 513.88 (Martens-Uzunova and Schaap, 2009). The presence of *pelE* in *A. niger* CBS 120.49 was described 30 years ago, although some doubt was raised about its actual function (Harmsen et al., 1990). The genomes of the *A. tubingensis* and *A. luchuensis* strains from which pectin lyases were sourced in the current work also contain *pelE* genes (UniProt entries A0A100I604 and G7XZW9, respectively), but no reports on PelE expression or activity of the pectin lyases from these strains exist. In a recent study, all five pectin lyase genes of *A. niger* CBS 513.88 – *pelA*, *pelB*, *pelC*, *pelD*, and *pelF* – were cloned and overexpressed in *A. niger* SH-2 (He et al., 2018). Of these, the *pelA*-recombinant strain exhibited the highest pectin lyase activity on apple pectin (DM 50–75%), and thus only PelA was selected for characterization.

In the current work, we aimed to express and systematically characterize representatives of *Aspergillus* sp. PelA, PelB, PelC, PelD, and PelF. The PL1_4 pectin lyases selected for the current work originated from three different strains which all belong to the *Aspergillus* section *Nigri*: *A. aculeatus, A. tubingensis*, and *A. luchuensis* (Table 1). The two latter strains were originally both assigned as *A. niger*, but subsequently determined via sequence analysis to in fact be the very closely related species *A. tubingensis* and *A. luchuensis*. Indeed, *A. tubingensis* and *A. luchuensis* both belong to the *A. niger* clade (Varga et al., 2011; Hong et al., 2013). We attempted to express *Al*PelA recombinantly in *A. oryzae*, but did not succeed. The PelA from *A. tubingensis* (which we named *At*PelA for the current work) is 99% identical to the PelA of *A. luchuensis* (*Al*PelA; UniProt G7XV55) and is therefore useful as a PelA representative to compare to PelB, PelC, PelD, and PelF from *A. luchuensis*. The UniProt entries list different *Aspergillus* species as origins of the employed pectin lyases, e.g., *A. kawachii* which has since been replaced by *A. luchuensis* (Hong et al., 2013). Together, these shifting taxonomic designations indicate that assigning exact *Aspergillus* species classification is a complex matter. In the current work, we have named the pectin lyases according to their most recent species classification as well as to their identity with the *A. niger* CBS 513.88 pectin lyases named PelA, PelB, PelC, PelD, and PelF (Table 1).

**FIGURE 2:**
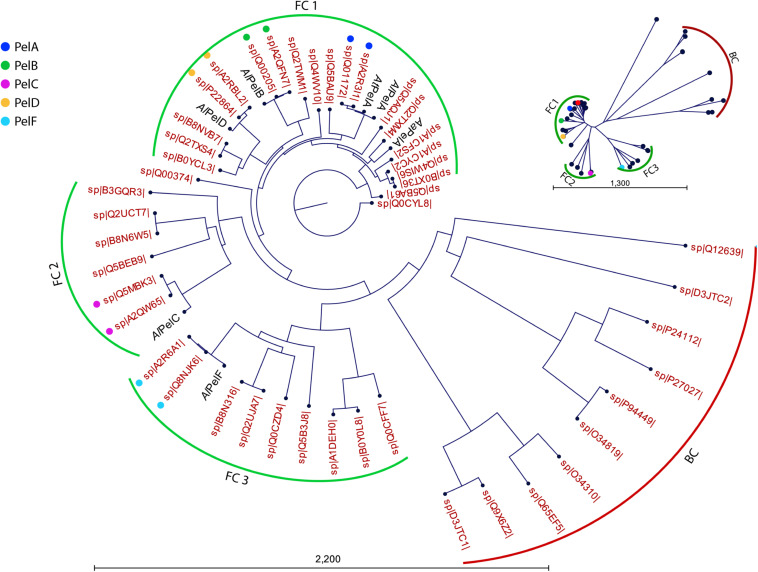
Phylogenetic tree of pectin lyases included in the current study shown versus all reviewed Swiss-Prot entries (red labels indicating Swiss-Prot accession numbers). From the radial tree (top right), three clusters were identified: a bacterial cluster (BC, red) and three fungal clusters (FC1-FC3, green). Pectin lyases PelA, PelB, PelC, PelD, and PelF from *A. niger* strains CBS 513.88 and CBS 120.49 are indicated with colored dots. The UniProt database does not indicate CBS 120.49 in these entries, but tracking the submissions they all originate from *A. niger* CBS 120.49. *Al*PelA from the same *A. luchuensis* strain as *Al*PelB, *Al*PelC, *Al*PelD, and *Al*PelF is indicated to visualize its close relatedness to *At*PelA studied here.

In order to allow comparison of the current work to literature, the six PL1_4 pectin lyases were compared to Swiss-Prot pectin lyase sequences in terms of sequence identity (Table 1) and phylogeny (Figure 2). A clear distinction between bacterial and fungal pectin lyases is observed (Figure 2). *At*PelA, *Aa*PelA, *Al*PelD, and *Al*PelB cluster in the same fungal cluster (FC1), whereas *Al*PelC and *Al*PelF cluster in each their fungal cluster (FC2 and FC3, respectively, Figure 2). This division is also evident from their pairwise sequence comparisons: *Al*PelC and *Al*PelF are clearly different from the members of FC1 and also different from each other (sequence identities ≤ 50%; Supplementary Figure S2). The putative PelE from *A. niger* CBS 120.49 (SwissProt B3GQR3) clusters at the edge of FC2 containing the PelC representatives, which have not been deeply characterized either (Figure 2 and Supplementary Table S1). The phylogenetic tree emphasizes the close relatedness of the pectin lyases used in the current work to those of *A. niger* CBS 513.88 and CBS 120.49 Figure 2); this is also apparent from their high sequence identity (Table 1). Furthermore, the high similarity between *At*PelA and PelA from *A. luchuensis* (*Al*PelA) is also clear from the phylogenetic analysis (Figure 2). As expected, *Aa*PelA clusters with *At*PelA and PelA (Figure 2, Table 1, and Supplementary Figure S2). The decision to include *Aa*PelA and no other *A. aculeatus* PL1_4 enzymes in the study reflects the fact that PelA is the most studied enzyme of the five, possibly due to its high activity and expression levels (He et al., 2018).

**FIGURE 3:**
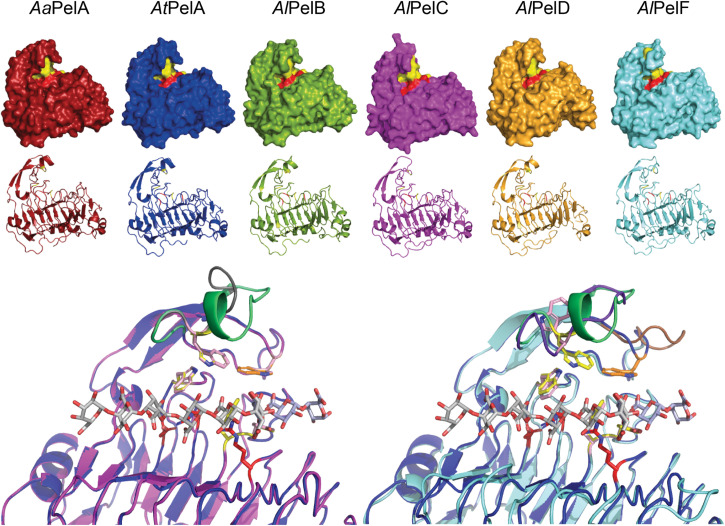
*Top:* Homology models of the six PL1_4 pectin lyases studied here. Three charged residues believed to hold the catalytic function (one Asp and two Arg residues) are indicated in red, while aromatic residues which interact with the substrate and maintain the active site architecture are indicated in yellow (Mayans et al., 1997; Herron et al., 2002; Van Alebeek et al., 2002). The C-terminals of *Al*PelD and *Al*PelF could not be modeled and are thus not visible. *Bottom:* Active site zoom in structural alignments of homology models of *At*PelA (blue) and *Al*PelC (magenta) and of *At*PelA and *Al*PelF (cyan), respectively. The pectate oligomer ligands (gray and light blue) are derived from alignment with *Bacillus subtilis* pectate lyase (PDB IDs: 3KRG and 5AMV) (Seyedarabi et al., 2010; Ali et al., 2015). Assumed catalytic residues (red) are indicated alongside substrate interacting residues (yellow in *At*PelA, light pink in *Al*PelC and *Al*PelF). The substrate-interacting Trp86 in *At*PelA (corresponding to Trp66 in crystal structures of *A. niger* PelA and PelB (Mayans et al., 1997; Vitali et al., 1998; Herron et al., 2002), which were numbered without the 20-amino acid signal peptide) is shown in orange to highlight its absence in *Al*PelF, where this differing loop region is shown in brown (Supplementary Figure S3). The loop above the active site, which is four amino acids shorter in *Al*PelC and *Al*PelF and contains the substrate-interacting Trp residue corresponding to Trp81 in *A. niger* PelA and PelB (Mayans et al., 1997; Vitali et al., 1998; Herron et al., 2002), is highlighted in green in *At*PelA, gray in *Al*PelC, and purple in *Al*PelF. Alignment of *Al*PelF and *Al*PelC with *Al*PelB is highly similar to that with *At*PelA (not shown).

Two PL1_4 subfamily members have resolved crystal structures: *A. niger* PelA (PDB IDs 1IDK and 1IDJ; Mayans et al., 1997), which has 99% sequence identity to *At*PelA, and *A. niger* PelB (PDB ID 1QCX; Vitali et al., 1998), which has 98% sequence identity to *Al*PelB. Structurally, the two pectin lyases appear highly similar. Neither contain ligand structures, but several crystal structures of bacterial PL1 pectate lyases from other subfamilies do. Together, this enables homology modeling and identification of the active site in the PL1_4 pectin lyases studied here. From the homology models it is evident that the three-dimensional structures of *Aa*PelA, *At*PelA, *Al*PelB, and *Al*PelD are indeed very similar (Figure 3). In contrast, *Al*PelC and *Al*PelF appear to exhibit a more open conformation around the active site and the homology models suggest that *Al*PelC and *Al*PelF have a more disordered and open loop in this position instead of the short α-helix observed in the crystal structures of PelA and PelB as well as in the models of *Aa*PelA, *At*PelA, *Al*PelB, and *Al*PelD (Figure 3). From the multiple sequence alignment it is evident that *Al*PelC and *Al*PelF have shorter loops in this region (marked in blue in Supplementary Figure S3). This loop contains a conserved substrate-interacting Trp residue corresponding to Trp81 in *A. niger* PelA and PelB (Mayans et al., 1997; Vitali et al., 1998; Herron et al., 2002). In the *Al*PelC homology model this Trp is completely aligned with that of *At*PelA, whereas the Trp side chain points in the opposite direction in the *Al*PelF model (Figure 3). For a neighboring loop above the active site (marked in gray in Supplementary Figure S3), *Al*PelC is similar to the other pectin lyases, whereas *Al*PelF exhibits a longer, disordered loop in this position (Figure 3). Importantly, this loop contains a Trp residue (Trp66 in *A. niger* PelA and PelB), which interacts with the methoxyl group of GalA in subsite +3 (Mayans et al., 1997; Herron et al., 2002). This Trp residue is generally conserved, but absent from *Al*PelF (Figure 3 and Supplementary Figure S3). Furthermore, *Al*PelF is larger than the other five pectin lyases, which are almost identical in predicted size (glycosylations excluded; Table 1). This larger size is due to a C-terminal stretch found in *Al*PelF (Supplementary Figure S3), which according to a BLAST analysis appears to be unique to PelF from various *Aspergillus* species. The function of this C-terminal stretch is unknown; it could not be modeled by homology modeling, and secondary structure prediction suggested the entire structure to be a random coil. In conclusion, *Al*PelF differs most from the generally similar structures of the other pectin lyases. This assessment of the six pectin lyases show that there is good agreement between the phylogenetic analysis and the three-dimensional enzyme structure (Figures 2, 3).

Using the CUPP webserver for functional annotation (Barrett and Lange, 2019; Barrett et al., 2020a) confirmed that all the included enzymes are predicted to belong to PL1_4. *Aa*PelA, *At*PelA, *Al*PelB, *Al*PelD, and *Al*PelF were all assigned EC 4.2.2.10 and CUPP group PL1:12.1^1^ (Barrett et al., 2020a). In contrast, *Al*PelC could not be assigned to a CUPP group due to a low score. Although *Al*PelF differs most in terms of structure (Figure 3), CUPP singled out *AlPelC* as being more different. The detailed grouping provided by CUPP relies on an unsupervised peptide-based clustering algorithm that can provide systematic grouping and functional annotation of carbohydrate-active enzymes (CAZymes) based on comparison of peptide motifs in the enzyme amino acid sequences (Barrett and Lange, 2019). This emphasizes that *Al*PelC may be functionally different from the other pectin lyases studied here.

### Characterization of Pectin Substrates

Quantitatively, the major constituent of pectin is homogalacturonan followed by rhamnogalacturonan I (RG-I). Homogalacturonan consists of an unbranched chain of α-(1,4)-linked GalA units, which may be methoxylated and/or acetylated. Homogalacturonan can also be further substituted to form other less abundant pectin components, namely xylogalacturonan, apiogalacturonan, and rhamnogalacturonan II (RG-II). RG-I is made up of a backbone of alternating α-(1,2)-linked rhamnose and α-(1,4)-linked GalA units, where the rhamnose units are substituted with neutral sidechains of galactan, arabinan, and/or different arabino-galactan sidechains (Bonnin et al., 2014).

Compared to apple and citrus pectins, sugar beet pectin is known to have shorter homogalacturonan stretches, more RG-I, and a higher degree of acetylation (DAc) (Buchholt et al., 2004). This was reflected in the monosaccharide composition observed in the current work: sugar beet pectin contained more rhamnose, arabinose, and galactose (indicators of RG-I) and less GalA than apple and citrus pectins (Table 2). The DAc was 19% for sugar beet pectin and only 1% and 2% for citrus and apple pectins, respectively (Table 2). Comparing apple and citrus pectins, citrus pectin had slightly more GalA. Since pectin lyase prefers methylesterified GalA, the degree of methoxylation (DM) is an important parameter: Apple pectin had the highest DM of 69%, whereas there was no significant difference between sugar beet pectin (56%) and citrus pectin (53%; Table 2). This is in agreement with previous reports on DM for these pectin sources (Müller-Maatsch et al., 2016). Not accounting for other factors such as methyl group distribution, the pectin lyases were thus expected to have the highest affinity for apple pectin due to the high DM. Similar DMs were observed for citrus pectin and sugar beet pectin, but due to the significantly higher DAc observed in sugar beet pectin, it was expected that the pectin lyases would exhibit the lowest affinity for sugar beet pectin.

### Enzyme Activity on Different Pectins

The specific activity of the six pectin lyases was determined as initial rates obtained at 25°C in 1 g/L solutions of apple pectin (DM 69%, DAc 2%), citrus pectin (DM 53%, DAc 1%), sugar beet pectin (DM 56%, DAc 19%), and polygalacturonic acid at pH 5.0 (Table 3). The highest initial rate was obtained with *Al*PelB on apple pectin. Indeed, all the pectin lyases exhibited highest activity on apple pectin, which is likely linked to the high DM of this pectin substrate. In general, the following substrate preference was observed: apple pectin > citrus pectin > sugar beet pectin > polygalacturonic acid, which reflects both DM and DAc of the substrates (Table 3). As expected, the difference in DM appeared to determine the difference in preference between apple and citrus pectins (Tables 2, 3), whilst the markedly higher DAc of sugar beet pectin perhaps in combination with a higher amount of RG-I (Table 2) impaired pectin lyase activity on sugar beet pectin. Finally, the consistently low activities of all pectin lyases on polygalacturonic acid (Table 3) confirm the requirement for methoxylation to achieve pectin lyase activity. The same trend was recently reported for PelA from *A. niger* CBS 513.88, which had highest activity on citrus pectin (DM ≥ 85%), a relative activity of 59% compared to citrus pectin on a sample of apple pectin with a DM of 50–75%, and almost no activity on polygalacturonic acid (He et al., 2018).

**TABLE 3:** Specific enzyme activity (μmol product released per min per mg enzyme) determined from initial rates of the six pectin lyases on four different pectin substrates at pH 5 and 25°C: Apple pectin, citrus pectin, sugar beet pectin, and polygalacturonic acid; c.f. Table 2 for composition of apple, citrus, and sugar beet pectins.

	Apple pectin	Citrus pectin	Sugar beet pectin	Polygalacturonic acid
*Aa*PelA	3.9 ± 0.2^d^	2.5 ± 0.1^e^	1.1 ± 0.05^f^	0.06 ± 0.03^i^
*At*PelA	8.7 ± 0.3^b^	2.4 ± 0.1^e^	0.7 ± 0.04^g^	0.07 ± 0.02^i^
*Al*PelB	9.1 ± 0.2^a^	5.2 ± 0.2^c^	0.8 ± 0.06^g^	0.06 ± 0.06^i^
*Al*PelC	≤0.0005^i^	≤0.0005^i^	≤0.0005^i^	≤0.0005^i^
*Al*PelD	0.34 ± 0.06^h^	0.08 ± 0.01^i^	0.008 ± 0.007^i^	0.03 ± 0.03^i^
*Al*PelF	0.06 ± 0.01^i^	0.02 ± 0.01^i^	0.04 ± 0.01^i^	0.02 ± 0.01^i^

*Superscript letters indicate significant difference (p < 0.05) across the entire dataset.*

Among the six pectin lyases, *Al*PelB had the highest initial rate on both apple pectin and citrus pectin, whereas *Aa*PelA had highest activity on sugar beet pectin (Table 3). *At*PelA had higher activity on apple pectin than *Aa*PelA, but the opposite was observed for sugar beet pectin (Table 3), and the two PelA pectin lyases exhibited similar activity on citrus pectin and polygalacturonic acid. For apple and citrus pectins, the following general ranking in specific activity was observed: PelB > PelA > > PelD > PelF > PelC (Table 3). The activity of *Al*PelC was at the detection limit despite using a high enzyme dosage. For polygalacturonic acid there was no significant difference between the very low specific activities, indicating that the enzymes do not have pectate lyase activity.

In a recent study, the fermentation broths of *A. niger* SH-2 used for recombinant expression of *A. niger* CBS 513.88 PelA, PelB, PelC, PelD, and PelF were tested on apple pectin (DM 50–75%) at pH 5.2. The enzyme activities in U/mL was PelA > PelD > > PelF > PelB, while PelC could not be expressed. However, these activities also reflect the expression levels, and especially PelB was expressed in lower amounts than the other pectin lyases (He et al., 2018). It was previously reported that *A. niger* CBS 120.49 PelB was more prone to protease degradation than PelA of the same strain when expressed homologously (Kusters-van Someren et al., 1992). As a consequence, it may not be possible to detect the high specific activity of PelB without controlling the enzyme dosage.

### pH-Temperature Optima and Thermal Stability

Data on pH and temperature optima of pectin lyases from *Aspergillus* sp. are scarce (Supplementary Table S1). Recently, the optima for PelA from *A. niger* CBS 513.88 were determined separately on citrus pectin (DM ≥ 85%) to pH 4.5 and 50°C (He et al., 2018). Previously, PelA from *A. niger* (identified as PLII in the commercial Ultrazym preparation) was found to have a slightly substrate concentration-dependent optimum around pH 5.5–6.5 when determined at 25°C on apple pectin with a DM of 94% (van Houdenhoven, 1975; Kester and Visser, 1994). The same was true for Ultrazym PLI, which was found to be PelD from *A. niger* (van Houdenhoven, 1975; Gysler et al., 1990; Kester and Visser, 1994). PelF from *A. niger* ZJF also exhibited an acidic pH optimum of 5.0 and a temperature optimum of 43°C on citrus pectin (Xu et al., 2015). In contrast, PelB from *A. niger* CBS 120.49 had an alkaline optimum at pH 8.5 when determined at 25°C (Kester and Visser, 1994).

As pH optima are temperature dependent, we set out to estimate combined pH-temperature optima for the four most active pectin lyase in the current study using response surface methodology. Based on preliminary studies, acidic pH ranges (pH 3.5–5.5) were selected for *At*PelA and *Al*PelD, whereas a range from slightly acidic to neutral pH (pH 5.5–7.5) was selected for *Aa*PelA and *Al*PelB. Temperatures ranged from 40°C to 70°C (Table 4) and the reaction was monitored for 5 min. All the generated models had *R*^2^ above 0.92, and most of them above 0.98, indicating that the modeled response surfaces described the data well (Supplementary Table S2 and Supplementary Figure S4). For most models, the lack of fit was statistically significant (Supplementary Table S2). This indicates that the generated models are not sufficient for predicting activity at the identified optima, and is a result of the fact that the model assumes that the effect of pH and temperature can be described by quadratic polynomials. This is, however, not always the case for pH and temperature optima, which often follow non-symmetrical bell-shaped curves. For a few of the models, one of the main factors (*T* or pH) was not significant, but they were kept in the model in order to predict a pH-temperature optimum for the four pectin lyases on three different pectins (Table 4, Supplementary Table S2, and Supplementary Figure S4). For *Aa*PelA on sugar beet pectin, no model was obtained as the differences between the data points in the design were not statistically significant within the chosen ranges (Supplementary Table S2 and Supplementary Figure S4).

**TABLE 4:** Combined pH-temperature optima on apple pectin, citrus pectin, and sugar beet pectin predicted from the regression models generated by the two-factor face-centered central composite experimental design (Supplementary Table S2).

	*T*_opt_ [°C]				pH_opt_			
	Test interval	Apple pectin	Citrus pectin	Sugar beet pectin	Test interval	Apple pectin	Citrus pectin	Sugar beet pectin
*Aa*PelA	45–65°C	55	54*	–	pH 5.5–7.5	6.4*	6.1*	–
*At*PelA	50–70°C	57*	58*	59*	pH 3.5–5.5	4.9*	4.5	4.4
*Al*PelB	40–60°C	40*	49*	48	pH 5.5–7.5	6.8*	6.4*	6.7*
*Al*PelD	40–70°C	59*	60	60	pH 3.5–5.5	4.9*	4.3*	4.4*

*An asterisk (^∗^) indicates that the main factor (T or pH) was statistically significant (p < 0.05) in this model. For AaPelA on sugar beet pectin, no model was obtained as the differences between the data points in the design were not statistically significant within the chosen ranges (Supplementary Table S2 and Supplementary Figure S4).*

In general, *At*PelA and *Al*PelD exhibited pH-temperature optima, which were a combination of high temperature and low pH: pH 4.4–4.9 and 57–59°C for *At*PelA and pH 4.3–4.4 and 59–60°C for *Al*PelD (Table 4). However, for *Al*PelD on citrus and sugar beet pectins, *T* was not a significant factor, indicating low variability across the range from 40 to 70°C (Supplementary Figure S4). The same was true for pH for AtPelA (Table 4 and Supplementary Table S2). For *Aa*PelA and *At*PelB, the pH-temperature optima were a combination of lower temperature and higher pH: pH 6.1–6.4 and 54–55°C for *Aa*PelA and pH 6.4–6.8 and ≤ 40–49°C for *Al*PelB (Table 4). These trends are fairly well in line with previously reported optima for *A. niger* pectin lyases (Supplementary Table S1), and can partially explain the pectin lyase multigenecity in *Aspergilli*. For *Al*PelB we observe a lower (yet high in comparison with the other pectin lyases) pH optimum than found for PelB from *A. niger* CBS 120.49 (Kester and Visser, 1994). This is most likely tied to the fact that we have included an elevated temperature (40–60°C), whereas the previously established pH optimum of 8.5 was determined at 25°C, where PelB is more stable. Remarkably, the fact that the two PelA pectin lyases included here differ in their pH-temperature optima emphasize that results obtained with a given pectin lyase from one *Aspergillus* strain is not directly transferable to one of another strain. However, this effect may not be as dramatic within the *A. niger* clade where sequence similarities above 90% are observed for the enzymes discussed here, whereas the sequence similarity between *Aa*PelA and *At*PelA is only 75% (Table 1 and Supplementary Figure S2).

Generally, pH values of the optima were higher on apple pectin than on citrus or sugar beet pectins (Table 4). The *pK*_a_ of pectin decreases with increasing pH (Plaschina et al., 1978). Possibly, the higher DM of the apple pectin entails that a higher pH is required to reach the net charge preferred by the pectin lyases as compared to the lower-DM pectins.

Thermal stability of the pectin lyases was assessed by determining the melting temperatures (*T*_m_) at pH 5. The highest *T*_m_ was found for *Aa*PelA (75°C), followed by *At*PelA (68°C), *Al*PelD (67°C), and *Al*PelB (65°C). The lowest *T*_m_ values were found for *Al*PelF (61°C) and *Al*PelC (57°C). Significant difference (*p* < 0.05) was observed between all values, indicating that the PelA pectin lyases were the most thermally stably, whereas the enzymes of low specific activity, i.e., *A*lPelF and *Al*PelC, also had the lowest thermal stability.

### Kinetic Parameters

The kinetic parameters of the four most active pectin lyases were determined at the estimated pH-temperature optima (Table 4) on apple pectin, citrus pectin, and sugar beet pectin (Table 5). The enzymes differed in terms of *K*_m_ values on the different substrates except for *Aa*PelA where no significant difference in *K*_m_ was observed across the three pectins (11–14 mM GalA). For *At*PelA the *K*_m_ was significantly lower on apple pectin (8 mM GalA) than on citrus pectin (19 mM GalA), and highest on sugar beet pectin (25 mM GalA). For both *Al*PelB and *Al*PelD, no significant difference in *K*_m_ was observed between citrus and sugar beet pectins, indicating that acetylations had little effect on affinity for these enzymes (Table 5). The effect of methoxylation differed between the two: while *K*_m_ decreased with increasing DM for *Al*PelB, an increase was observed for *Al*PelD (Table 5). The lowest *K*_m_ values were observed for *At*PelA on apple pectin and for *Al*PelD on citrus and sugar beet pectins (Table 5). The latter were at the same level as that observed previously for *A. niger* PelD on high-methoxylated apple pectin (Supplementary Table S1). However, for *A. niger* PelA and PelB, previously reported *K*_m_ values are lower than the values observed here; this could be linked to the use of chemically esterified pectins in previous studies, where the pectins were esterified to a much higher DM than we used here, namely a very high DM of around 95% (Supplementary Table S1) (van Houdenhoven, 1975; Kusters-van Someren et al., 1991; Kester and Visser, 1994).

**TABLE 5:** Kinetic parameters *K*_m_ (in mM GalA) and *k*_cat_ (in mol product released per mol enzyme per second) for *Aa*PelA, *At*PelA, *Al*PelB, and *Al*PelD on apple pectin (DM 69 ± 3%, DAc 2 ± 0.1%), citrus pectin (DM 53 ± 1%, DAc 1 ± 0.1%), and sugar beet pectin (DM 56 ± 5%, DAc 19 ± 1%).

Enzyme	Substrate	pH	*T*	*R*^2^	*K*_m_	*k*_cat_	*k*_cat_/*K*_m_
					
			°C		mM	s^–1^	mM^–1^ s^–1^
*Aa*PelA	Apple pectin	6.4	55	1.00	110.4^*x*,*a*^	872.8^*x*,*c*^	7.60.01^*x*,*c*^
	Citrus pectin	6.1	54	0.99	140.8^*x*,*c*^	541.6^*y*,*c*^	3.80.1^*y*,*c*^
	Sugar beet pectin	5.5	55	0.92	132.5^*x*,*b*^	173.1^*z*,*c*^	1.30.1^*z*,*c*^
*At*PelA	Apple pectin	4.9	57	0.99	7.91.4^*z*,*b*^	13611^*x*,*b*^	172^*x*,*b*^
	Citrus pectin	4.5	58	0.99	191.8^*y*,*b*^	1318.5^*x*,*b*^	7.00.2^*y*,*b*^
	Sugar beet pectin	4.4	59	0.96	254.0^*x*,*a*^	488.4^*y*,*b*^	1.90.1^*z*,*b*^
*Al*PelB	Apple pectin	6.8	40	0.98	132.7^*y*,*a*^	27435^*x*,*a*^	222.7^*x*,*a*^
	Citrus pectin	6.4	49	1.00	231.8^*x*,*a*^	29418^*x*,*a*^	130.2^*y*,*a*^
	Sugar beet pectin	6.7	48	0.91	211.6^*x*,*a*^	963.6^*y*,*a*^	4.50.3^*z*,*a*^
*Al*PelD	Apple pectin	4.9	59	0.98	130.9^*x*,*a*^	140.3^*x*,*d*^	1.10.1^*x*,*d*^
	Citrus pectin	4.3	60	0.99	8.90.7^*y*,*d*^	8.70.2^*y*,*d*^	1.00.1^*x*,*d*^
	Sugar beet pectin	4.4	60	0.90	81.5^*y*,*c*^	2.60.1^*z*,*d*^	0.30.1^*y*,*d*^

*Reactions were performed at the estimated pH-temperature optima (Table 4). The R^2^ values for the linear regression on the Hanes-Woolf plots are indicated, and reactions were run in triplicates. Superscript letters a-d indicate significant difference (p < 0.05) between the values of each parameter for the four enzymes when compared on the same substrate, whereas superscript letters x-z indicate significant difference (p < 0.05) between the three substrates for the same enzyme.*

For all enzymes, *k*_cat_ was significantly lower on sugar beet pectin than on the other pectin substrates (Table 5). For *Aa*PelA and *Al*PelD, *k*_cat_ was highest on apple pectin, whereas there was no significant difference between *k*_cat_ values on apple and citrus pectins for *At*PelA and *Al*PelB (Table 5). For *Aa*PelA, *At*PelA, and *Al*PelB *k*_cat_/*K*_m_ was significantly higher on apple pectin. For *Al*PelD the difference to citrus pectin was not statistically significant, but the value for apple pectin was higher. For all enzymes, *k*_cat_/*K*_m_ was significantly lower on sugar beet pectin (Table 5). Again, this directly reflects the preference of pectin lyases for a highly methoxylated substrate and suggests a preference for substrates with low DAc. Regardless of the substrate, large differences in *k*_cat_/*K*_m_ were observed between the enzymes, which ranked in the following order with respect to *k*_cat_/*K*_m_: *Al*PelB > *At*PelA > *Al*PelA > *Al*PelD (Table 5). The finding that *Al*PelB was the most efficient enzyme is in good agreement with literature data on *A. niger* pectin lyases, where the highest *k*_cat_/*K*_m_ has indeed been observed for PelB (Supplementary Table S1). In that previous study (Kester and Visser, 1994), the *k*_cat_/*K*_m_ was higher than observed in the current work, which could be due to the use of a highly methylesterified substrate, and that the reaction took place at pH 8.5. However, such a high pH is close to the pH where pectin spontaneously decomposes by β-elimination (Renard and Thibault, 1996). Analogously, significantly lower *k*_cat_/*K*_m_ values were obtained for *Aa*PelA and *Al*PelD as compared to the *A. niger* PelA and PelD, which for *Aa*PelA was linked to an eight times higher *K*_m_ and for *Al*PelD to a five times higher *k*_cat_ (Table 5 and Supplementary Table S1). For *At*PelA the *k*_cat_/*K*_m_ of 17 mM^–1^ s^–1^ on apple pectin was comparable to that of the *A. niger* PelA (18–22 mM^–1^ s^–1^); the use of a higher reaction temperature in the current work to increase *k*_cat_ balanced out the negative effect of the lower DM on *K*_m_ in this particular case (Table 5 and Supplementary Table S1).

### Pectin Degradation Product Profiling by LC-ESI-MS and SEC

**FIGURE 4:**
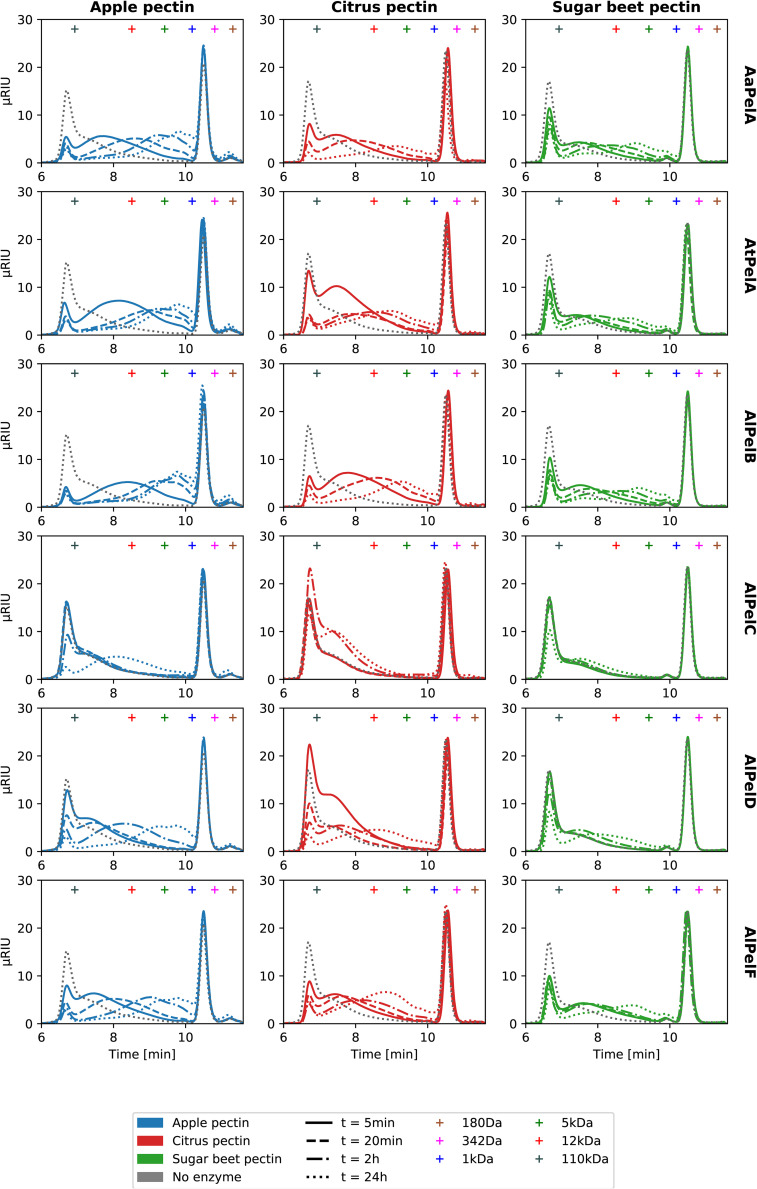
Size exclusion chromatography (SEC) chromatograms for degradation of 10 g/L apple pectin (blue), citrus pectin (red), and sugar beet pectin (green) by 60 nM *Aa*PelA, *At*PelA, *Al*PelB, or *Al*PelD (corresponding to 0.023% E/S for *At*PelA) and 600 nM *Al*PelC or *Al*PelF at pH 5.5 and 40°C. For chromatograms of *Al*PelC and *Al*PelF at 60 nM, refer to Supplementary Figure S5. Retention times of pullulan standard (180 Da to 110 kDa) are indicated by crosses.

To compare the pectin degradation profiles of all six pectin lyases, they were dosed in equimolar ratios on apple pectin, citrus pectin, and sugar beet pectin. For simplicity, a single set of reaction conditions was selected, reflecting the best compromise based on the obtained pH-temperature optima (Table 4): pH 5.5 and 40°C. The reactions were monitored over 24 h and analyzed by size exclusion chromatography (SEC) and liquid chromatography coupled to mass spectrometry with electrospray ionization (LS-ESI-MS). From the SEC chromatograms, the concentration of pectin molecules below ~100 kDa could be monitored over time (Figure 4). The SEC analysis of pectin degradation products reflect the enzyme activity and substrate specificity data well: Apple pectin is degraded faster than citrus pectin, which is in turn degraded faster than sugar beet pectin (Figure 4). In a comparison of the six pectin lyases, the specific activities (Table 3) are directly reflected in the development of the SEC profiles over time: PelB > PelA > PelD > PelF > PelC (Figure 4 and Supplementary Figure S5). Comparing the two PelA pectin lyases, they were again found to be equally fast on citrus pectin, while *At*PelA was faster on apple pectin, and *Aa*PelA was faster on sugar beet pectin (Figure 4 and Table 3). As a consequence of their low activity, *Al*PelC and *Al*PelF were dosed 10 times higher than the other pectin lyases in order to better capture their product profiles in the LC-MS-based product profiling. For *Al*PelF the increased dosage had a large effect on pectin degradation (Supplementary Figure S5). For *Al*PelC the very low activity was only detectable after 24 h, with a noticeable difference between the two enzyme dosages (Figure 4 and Supplementary Figure S5).

The product profiles obtained by LC-ESI-MS were expected to contain both partly and fully methoxylated structures. Partly methoxylated pectin-derived oligosaccharides can easily be identified by MS^2^ in negative mode (Quéméner et al., 2003a; Ralet et al., 2005; Leijdekkers et al., 2011; Remoroza et al., 2012), whereas fully methoxylated pectin derived oligosaccharides can be identified in positive mode (Van Alebeek et al., 2000; Mutenda et al., 2002; Quéméner et al., 2003b). Manual inspection of the chromatograms obtained in negative mode revealed 28 unique compounds and 5 isomers all present in at least one enzyme-substrate-time combination (Table 6). The nomenclature of the identified compounds are gA_x_m_y_a_z_, meaning x GalA residues, y methyl substitutions, and z acetyl substitutions, e.g., a trimer with three GalA, one methyl substitution, and one acetyl substitution is denoted gA_3_m_1_a_1_. All compounds observed in negative mode appeared as [M-H], except gA_3_m_3_ and gA_4_m_4_ which appeared as formate [M+FA] ^–^ adducts and with a disproportionally low intensity. For all compounds observed in single charge state, the molecular formula could be confirmed by MS^2^ fragmentation with almost exclusively C*_i_* (or Z*_j_* due to isomeric masses) and ^0,2^A*_i_* fragments observed, according to the nomenclature of Domon and Costello (Domon and Costello, 1988), including the occasional loss of 32 Da that can be attributed to methoxylation at the galacturonic acid residue in the reducing end (Supplementary Figure S6; Quéméner et al., 2003b). For larger structures, e.g., molecular masses larger than ~1100 Da, a tendency toward double charging was observed (Table 6), making full identification from the fragmentation pattern difficult. Hence, for these compounds it was necessary to rely on the *m*/*z* values only. All compounds carried a 4,5 unsaturation in the non-reducing end due to the lyase reaction. For some masses, two distinct peaks were observed with two different retention times, indicating two different molecular structures (denoted #1 or #2; Table 6). The different retention times of the compounds are assumed to derive from different positions of the methyl and/or acetyl substitutions. The porous graphite column in use has previously shown the capability to separate different isomers of compounds with identical molecular composition, but with different linkages and/or planarity (Jamek et al., 2018; Mosbech et al., 2018; Zeuner et al., 2018).

**TABLE 6:** Compound ID, molecular weight (MW), number of GalA residues, number of methyl- and acetyl substitutions, charge state, and observed *m*/*z* in positive mode of all identified pectic oligosaccharides.

Compound ID	MW	GalA	Methyl	Acetyl	Charge *z*	Observed *m*/*z*
gA_2_m_1_	366	2	1		+1	384
gA_2_m_1_a_1_	408	2	1	1	+1	426
gA_2_m_2_a_1_*	422	2	2	1	+1	440
gA_3_m_1_ #1	542	3	1		+1	560
gA_3_m_1_ #2	542	3	1		+1	560
gA_3_m_2_ #1	556	3	2		+1	574
gA_3_m_2_ #2	556	3	2		+1	574
gA_3_m_2_a_1_ #1	598	3	2	1	+1	616
gA_3_m_2_a_1_ #2	598	3	2	1	+1	616
gA_3_m_2_a_2_	640	3	2	2	+1	658
gA_3_m_3_	570	3	3		+1	588
gA_3_m_3_a_1_*	612	3	3	1	+1	630
gA_3_m_3_a_2_*	654	3	3	2	+1	672
gA_4_m_2_ #1	732	4	2		+1	750
gA_4_m_2_ #2	732	4	2		+1	750
gA_4_m_2_a_1_	774	4	2	1	+1	792
gA_4_m_2_a_2_	816	4	2	2	+1	834
gA_4_m_3_	746	4	3		+1	746
gA_4_m_3_a_1_ #1	788	4	3	1	+1	806
gA_4_m_3_a_1_ #2	788	4	3	1	+1	806
gA_4_m_3_a_2_	830	4	3	2	+1	848
gA_4_m_4_	760	4	4		+1	778
gA_4_m_4_a_1_ #1*	802	4	4	1	+1	820
gA_4_m_4_a_1_ #2*	802	4	4	1	+1	820
gA_4_m_4_a_2_*	844	4	4	2	+1	862
gA_5_m_3_	922	5	3		+2	479
gA_5_m_3_a_1_	964	5	3	1	+1/+2	982/500
gA_5_m_3_a_2_	1006	5	3	2	+2	521
gA_5_m_4_	936	5	4		+1/+2	954/486
gA_5_m_4_a_1_	978	5	4	1	+2	507
gA_5_m_4_a_2_	1020	5	4	2	+2	528
gA_5_m_5_*	950	5	5		+1/+2	968/493
gA_5_m_5_a_1_*	992	5	5	1	+2	514
gA_6_m_3_	1098	6	3		+2	567
gA_6_m_4_	1112	6	4		+2	574
gA_6_m_4_a_1_	1154	6	4	1	+2	595
gA_6_m_4_a_2_*	1196	6	4	2	+2	616
gA_6_m_5_	1126	6	5		+2	581
gA_6_m_5_a_1_*	1168	6	5	1	+2	602
gA_6_m_5_a_2_*	1210	6	5	2	+2	623
gA_6_m_6_*	1140	6	6		+2	588
gA_7_m_4_	1288	7	4		+2	662
gA_7_m_5_	1302	7	5		+2	669
gA_7_m_6_*	1335	7	6		+2	676
gA_7_m_6_a_1_	1358	7	6	1	+2	697
gA_7_m_7_*	1330	7	7		+2	683
gA_8_m_6_	1492	8	6		+2	764
gA_8_m_7_*	1506	9	6		+2	771

*An asterisk (^∗^) indicates compounds only observed in positive mode.*

Investigation of the chromatograms obtained in positive mode revealed another 14 unique compounds and 1 isomer all present in at least one enzyme-substrate-time combination (Table 6), with the majority being fully methoxylated compounds. All identified compounds were observed as single charge [M+NH_4_]^+^ or double charge [½M + NH_4_]^2+^ ammonium adducts due to the constant presence of ammonium formate in the eluent. The structural verification by MS^2^ fragmentation was somewhat complex due to an unexpected fragmentation pattern. From literature, fully methoxylated pectin derived oligosaccharides are expected to form C*_i_*/Z*_j_* ions upon fragmentation in positive mode (Mutenda et al., 2002), although a mixed pattern including all possible ion types can be observed for pectic oligosaccharides in general (Körner et al., 1999). In the present case, assuming B*_i_* and C*_i_* fragments and the NH_4_^+^ ion retained on all fragments, all observed fragments had and *m*/*z* value 7 Da lower than expected. For example, this pattern was observed in positive mode for gA_4_m_3_, for which the structure could be verified in negative mode (Supplementary Figures S6, S7A). A number of putative, fully methoxylated structures were observed in positive mode, including gA_3_m_3_, gA_4_m_4_, and gA_5_m_5_. They all shared the “B*_i_*/C*_i_* -7 Da” pattern (Supplementary Figures S7B–D). The compound gA_5_m_5_ was observed both as a single charge and a double charge ion, with the fragmentation pattern of the latter giving more information (Supplementary Figure S7E). Despite not being the most dominant fragments, true C*_i_* ions were observed. Since this pattern is observed for compounds where additional structural verification in negative mode is available, it is assumed that the proposed structures with full methoxylation are correct.

**FIGURE 5:**
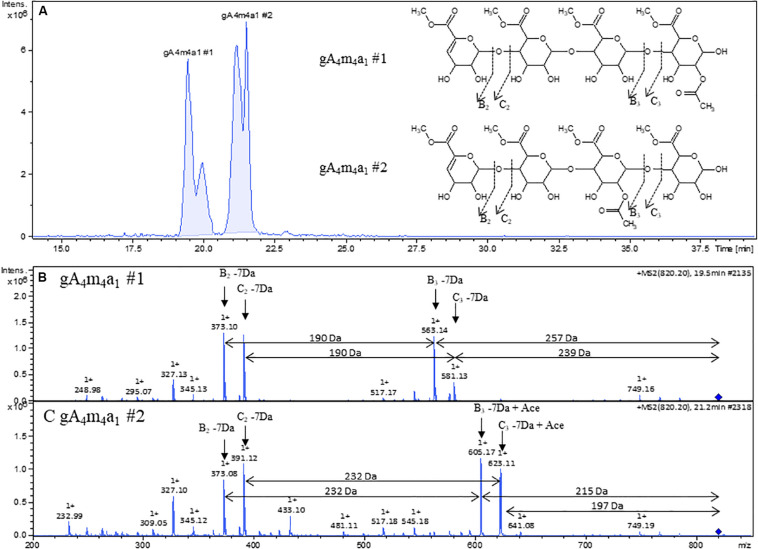
**(A)** Extracted ion chromatogram of 820 *m*/*z* with proposed structures inserted. **(B)** Positive ion mode ESI-MS^2^ of gA_4_m_4_a_1_ #1. **(B)** Positive ion mode of ESI-MS^2^ of gA_4_m_4_a_1_.

Contrary to fragmentation in negative mode, where only C*_i_* type (and isomeric Z*_j_* type) ions are observed, fragmentation in positive mode revealed additional information on acetylation patterns for different isomers sharing the same mass such as gA_4_m_4_a_1_ #1 and #2. The extracted ion chromatogram with the corresponding mass 820 *m*/*z* clearly indicates at least two different compounds with baseline separation (Figure 5A). The fragmentation pattern of gA_4_m_4_a_1_ #1 (Figure 5B) shows B_3_ and C_3_ fragments that have lost the acetyl group, indicating that the acetyl substitution is on the reducing end galacturonic acid unit. In contrast, the acetyl group is retained on B_3_ and C_3_ fragments and lost on B_2_ and C_2_ fragments in the fragmentation pattern of gA_4_m_4_a_1_ #2 (Figure 5C), indicating that the acetyl group is on the third galacturonic acid moiety when counting from the non-reducing end. No cross ring fragmentation was observed in either cases, leaving the position (O2 or O3) of the acetyl substitution unsolved.

The observed peak intensities showed that relatively few compounds, primarily gA_3_m_2_ and gA_3_m_3_, and to some extend gA_2_m_1_, gA_4_m_3_, gA_4_m_4_, and gA_6_m_4,_ were the dominant products for the *Aspergillus* sp. pectin lyases acting on apple and citrus pectins, with a more disperse profile when acting on sugar beet pectin (Supplementary Figure S8). An increased level of most of the identified compounds was observed over time, although with different rates dependent on type of compound, substrate and enzyme. The levels of larger structures such as gA_6_m_5_, gA_6_m_6_, gA_7_m_7_, and gA_8_m_7_ exhibited transient maxima during the 24 h reaction, indicating that these pectic oligomers were substrates for the pectin lyases (Supplementary Figure S8). The large variations in production rates among the enzymes are coherent with activity data (Table 3, Figure 4, and Supplementary Figure S8). Because of the large variation in activity among the enzymes, it conceals the details of the profiles for enzymes with lower activity, such as *Al*PelC and *Al*PelD. Hence, the data was normalized in order to facilitate an in-depth investigation and comparison of the product profiles.

**FIGURE 6:**
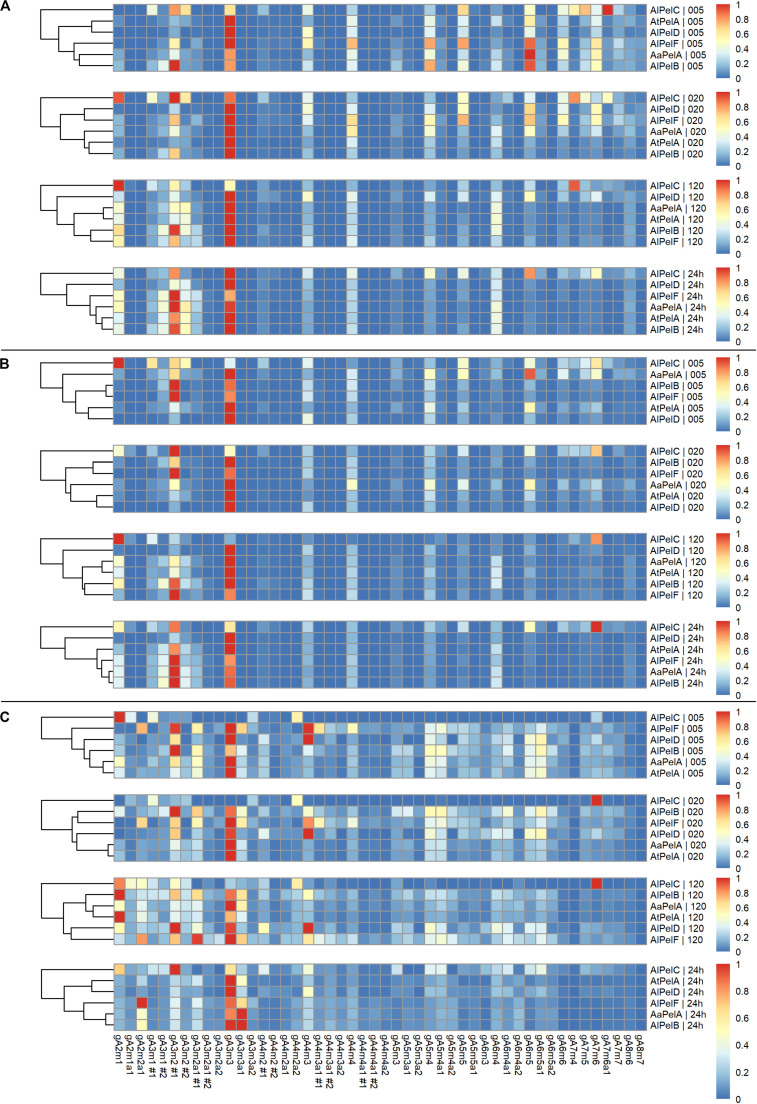
Heat maps of normalized relative intensities for pectin lyases acting on apple pectin **(A)**, citrus pectin **(B)**, and sugar beet pectin **(C)**. Panels top to bottom: 5, 20, 120 min, and 24 h of reaction. For each substrate the pectin lyases are clustered according to product profiles within each time point.

For all enzymes acting on apple pectin, gA_3_m_3_ was generally the most dominant product throughout the time course (Figure 6A). In the early stages (5, 20 min) some of the longer compounds were more highly represented (degree of polymerization (DP) of 5–7, especially gA_6_m_5_), but the relative abundance of these went down in favor of shorter compound like gA_3_m_2_ during the extended reaction (120 min, 24 h). The relative levels of gA_4_m_3_ and gA_4_m_4_ were steady throughout the time course, and there was a tendency to build-up of gA_6_m_4_ (Figure 6A). The shift from longer to shorter compounds was most predominantly observed for *Al*PelB, which has the highest specific activity (Table 3) and the highest *k*_cat_/*K*_m_ on apple pectin (Table 5). The product profilse after 24 h were quite similar for *Aa*PelA, *At*PelA, *Al*PelB, and *Al*PelF with gA_3_m_2_ and gA_3_m_3_ as the main products. An even narrower product profile was observed for *Al*PelD, clearly favoring gA_3_m_3_ and indicating a preference for fully methoxylated pectin. In contrast, a more dispersed profile was observed for *Al*PelC (Figure 6A). This more dispersed profile could be caused by the low activity of *Al*PelC (Table 3 and Figure 4), since the profile after 24 h looked more like the profiles of *Al*PelD and *Al*PelF after 20 min. However, it may also reflect a more selective substrate specificity, where the enzyme failed to degrade the longer compounds. Indeed, a vast increase in gA_6_m_5_ levels was observed for *Al*PelC during 24 h, whereas slight decreases in the observed peak intensities were observed for the other pectin lyases over the course of the reaction (Supplementary Figure S8). The clustering analysis of the product profiles was most clear after 24 h, with the profiles of *Al*PelB and *Al*PelA clustering closest together, and the profiles of *Al*PelD and *Al*PelC clustering second-most and most apart from the other pectin lyases, respectively.

In general, the product profile for all pectin lyases acting on citrus pectin looked similar to the profile on apple pectin (Figure 6B). This was expected due to the similarities of the two substrates in terms of fairly high methylation, low acetylation, and a low content of RGI. Similar product profile patterns were observed with gA_3_m_2_ and gA_3_m_3_ as the main end products for *Aa*PelA, *At*PelA, *Al*PelB and *Al*PelF, almost solely gA_3_m_3_ for *Al*PelD, and a more dispersed profile for *Al*PelC (Figure 6B). A notable difference compared to the profiles of enzymes acting on apple pectin was the lower relative levels of DP6-7 observed for citrus pectin. For apple pectin, especially gA_6_m_5_ was major contributor to the profiles particularly in the early stages of the reaction; a similarly, important contribution from this compound to the product profile was not observed on citrus pectin (Figures 6A,B). This might be due to the different distributions of methyl substitutions in the two substrates. The clustering analysis was almost identical to that observed for apple pectin, with the product profiles of *Al*PelB and *Aa*PelA clustering most, and the product profiles of *Al*PelD and *Al*PelC being the most different from the other enzymes after 24 h (Figure 6B).

From activity measurements (Tables 3, 5 and Figure 4) as well as the peak intensities of the individual compounds (Supplementary Figure S8), it is evident that sugar beet pectin is an inferior substrate compared to apple and citrus pectins, but decent degradation was nevertheless observed. The product profile of pectin lyases acting on sugar beet pectin was more differentiated than the profiles of apple and citrus pectins, due to the relatively higher presence of acetyl substituted compounds (Figure 6C). For all enzymes, except *Al*PelC, the major compound throughout the time course was gA_3_m_3_, with relatively high levels of DP5-6. The low levels of gA_6_m_6_ and longer compounds could be due to the nature of the sugar beet pectin substrate, where the structures necessary for releasing these products are present in limited amounts. The tendency of the longer compounds (DP5-6) to be outnumbered by the shorter compounds over time was also evident for the enzymes acting on sugar beet pectin. Remarkably, for *Aa*PelA and *Al*PelB one of the most dominating compounds was the fully methoxylated gA_3_m_3_a_1_, a compound that features a galacturonic acid residue with both a methyl and an acetyl substitution (Figure 5). These double substitutions has previously been reported as rare in sugar beet pectin (Ralet et al., 2005). The relative contribution of gA_3_m_3_a_1_ to the product profile compared to gA_3_m_3_ was most pronounced after 24 h, indicating that while especially *Aa*PelA, *Al*PelB, and *Al*PelF accommodate the acetylated, fully methoxylated substrate, they release the non-acetylated variant first (Figure 6C and Figure Supplementary S8). Again, a more dispersed profile was observed for *Al*PelC, with gA_3_m_2_ being the main product. The cluster analyses after 24 h were similar to the ones of apple and citrus pectins with the product profiles of *Al*PelB and *Aa*PelA clustering most, and *Al*PelC being most different (Figure 6C).

**FIGURE 7:**
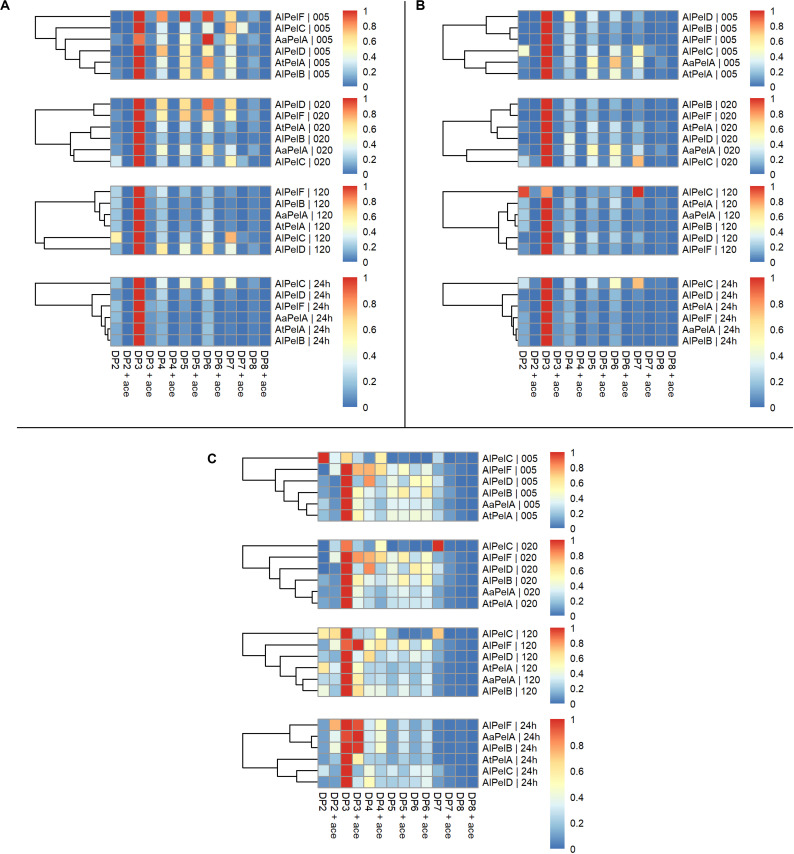
Heat maps of normalized summed relative intensities for individual degrees of polymerization divided non-acetyl substituted (DP columns) and acetyl substituted (DP ace columns) of pectin lyases acting on apple pectin **(A)**, citrus pectin **(B)**, and sugar beet pectin **(C)**. Panels top to bottom: 5, 20, 120 min, and 24 h. For each substrate the pectin lyases are clustered according to product profiles within each time point.

Examining the product profiles in a broader perspective with the intensities summed up according to DP with and without acetyl substitutions it was evident that DP3 is the most preferred product throughout the reactions (Figure 7). *Al*PelC showed a more differentiated profile compared to the other enzymes during the early phases of the reaction, but after 2 h of reaction DP3 was also dominant for this enzyme (Figure 7). In general, only negligible amounts of acetyl substituted compounds were observed in reactions on apple and citrus pectins, which was highly expected due to the low DAc of these substrates (Table 2), whereas slightly more differentiated profiles were observed for sugar beet reactions due to substrate acetylation. Hence, the division is more applicable for the profiles of enzymes acting on sugar beet pectin and the segregation revealed a division of the enzymes in two groups. *Aa*PelA, *Al*PelB, and *Al*PelF showed equal preference/tolerance for DP3 with and without acetyl substitutions as a product, and even a higher preference for releasing acetyl substituted DP4-6 compared to non-substituted DP4-6 (Figure 7). In contrast, *At*PelA, *Al*PelC, and *Al*PelD showed a notable preference for releasing non-acetylated DP3 compared to acetylated DP3. However, while *Al*PelC specifically preferred release of non-acetylated pectic oligosaccharides regardless of DP, the result was more blurred for *At*PelA and *Al*PelD, where the tolerance to acetylation changed across the DP range (Figure 7).

A broad product profile was observed after 5 and 20 min for all enzymes, with a shift toward the narrow profile of mainly DP3 over the 24 h of reaction. Even though a limited decrease in the levels of the longer DPs was observed (Supplementary Figure S8), this shift in the profiles can be explained by the production rates of the different compounds. The apparent production rate of DP3 is the sum of products originating from degradation of longer oligomers/polymers not accounted for in the profile analysis. It seems likely that DP3 does not serve as a substrate for any of the pectin lyases, which is in agreement with previous studies (Van Alebeek et al., 2002). In contrast, the apparent formation rate of longer DP, e.g., DP6, is the net sum of the positive production rate and the negative degradation rate. If the latter is lower than the former, the net result is a positive apparent production rate of DP6, albeit lower than the production rate of DP3. In all cases, a shift in the profiles from higher DP (DP5-7) to DP3 was observed, with the exception of *Al*PelC that had a more differentiated profile throughout the reactions, regardless of substrate (Figures 6, 7). For the cluster analyses on citrus and sugar beet pectins after 24 h reaction, *Aa*PelA and *Al*PelB clustered closest together, whereas *At*PelA and *Al*PelB clustered closest together in reactions on apple pectin (Figures 6, 7). Furthermore, *Al*PelD and *Al*PelC had the second and most different product profile, when compared to the rest of the enzymes after 24 h. Phylogenetically, *Al*PelF clustes far from PelA and PelB (Figure 2) and has a different profile than these enzymes at short reaction times (Figures 6, 7). However, at extended reaction times (and high enzyme dosage), the *Al*PelF profile became increasingly similar to that of the PelAs and *Al*PelB. Thus, the differences observed for *Al*PelF could merely be a result of low activity, which could in turn be explained by lack of an otherwise conserved substrate-interacting Trp residue (Figure 3).

### A Basis for Understanding Pectin Lyase Multigenecity in *Aspergilli*

Major differences in specific activity were observed among the different pectin lyases (Table 3), and through systematic experiments, we also observed differences in pH-temperature optima for PelA, PelB, and PelD (Table 4). Clearly, from a competitive growth perspective, the ability of the fungus to produce a battery of similar enzymes having optimal activity at different pH and temperature represents a major advantage, and this fitness theory provides an explanation for the multigenecity phenomenon also for the pectin lyases. Interestingly, the ranking of *k*_cat_/*K*_m_ across the three pectin substrates was the same for all of the four most active enzymes, but some discrepancies were observed for the *K*_m_ values, suggesting different propensity toward methyl and acetyl substitutions among the pectin lyases (Table 4). This interpretation was further substantiated by the detailed LC-MS product profiling, where differences in substrate degradation proficiency as well as in the end product profiles were observed between the enzymes (Figures 6, 7). In particular, the discriminative substrate degradation pattern analysis revealed a significant differential activity sensibility to acetyl substitutions.

*A. niger* regulates the expression of carbohydrate-degrading enzymes at the transcriptional level. At least three different transcriptional activators are linked to pectin degradation, namely GaaR, RhaR, and AraR, which control the production of enzymes responsible for degradation of homogalacturonan, RG-I, and RG-I arabinan and arabinogalactan side chains, respectively (Kowalczyk et al., 2017). Moreover, the GalA-dependent transcriptional repressor GaaX and the general carbon catabolite repressor CreA also take part in controlling expression of pectinolytic enzymes in *A. niger* including the pectin lyases (Kowalczyk et al., 2017; Niu et al., 2017). When grown on sugar beet pectin, PelA was found to be regulated by GaaR, whereas PelD was regulated by both GaaR, RhaR, and possibly also AraR (Alazi et al., 2016; Kowalczyk et al., 2017). These transcription regulators are proteins which are activated by the presence and concentration of certain monosaccharides or a catabolic product thereof (Kowalczyk et al., 2017). Indeed, GaaR is induced by 2-keto-3-deoxy-L-galactonate, an intermediate of the GalA catabolic pathway (Alazi et al., 2016, 2017). Signal molecules such as GalA may be released from the complex carbohydrates by enzymes which are constitutively expressed at low levels. Indeed, *A. niger* PelB, PelC, and PelF and several other pectinolytic enzymes were found not to be regulated by GaaR. Data indicated that they may instead be regulated by the general carbon catabolite repressor CreA and could therefore play a role in release of GalA to induce expression of PelA and PelD (de Vries et al., 2002; Niu et al., 2015; Kowalczyk et al., 2017). For PelE from *A. niger* CBS 120.49 transcription levels were very low, and it is therefore hard to conclude whether PelE is also regulated by the GalA-induced GaaX-GaaR system (Alazi et al., 2018). Expression profiling and transcriptomics on *A. niger* grown on sugar beet pectin or GalA revealed that PelA and PelD (both regulated by GaaR) were predominantly expressed/transcribed after short incubation times. In contrast, levels of PelB and PelF (mRNA) increased throughout 24 h of incubation (de Vries et al., 2002; Alazi et al., 2016). The mRNA levels of PelC were very low, again questioning its role in pectin degradation (Alazi et al., 2016, 2018), in agreement with the very low activity observed in the current work. Comparing mRNA levels a general tendency was observed: PelA > PelF > PelD > PelB, and PelC and PelE were hardly transcribed (Martens-Uzunova and Schaap, 2009; Alazi et al., 2016, 2018; Kowalczyk et al., 2017).

Based on this, PelA appears to remain the major pectin lyase responsible for pectin degradation by *Aspergilli* in nature with high activity and high expression levels. However, the fact that *Al*PelB was observed to be more efficient enzyme in the current work, indicates a possible important role of this enzyme in releasing GalA to induce the transcription of PelA despite low transcription levels, especially at elevated pH. Indeed, varying expression levels may be the reason for the presence of the much less efficient *Al*PelF to release GalA for GaaR activation. From an industrial perspective, where enzymes are produced recombinantly, *Al*PelB thus appears to be promising candidate for an efficient enzyme with a broad pH range. In contrast, the reason for maintaining *pelC* and *pelE* in the chromosomes is more elusive, given the inferior activity of AlPelC and low transcription levels of both enzymes. The fact that *pelE* could not be identified in *A. niger* CBS 513.88 (Pel et al., 2007; Martens-Uzunova and Schaap, 2009) may suggest evolutionary pressure to lose such genes from the multigene family.

## Conclusion

In order to study the multigenecity of pectin lyases in *Aspergilli*, representatives of PelA, PelB, PelC, PelD, and PelF were recombinantly expressed and characterized. A comparison of the pectin lyases in terms of activity at acidic pH, which is relevant for e.g., juice processing applications, the following ranking materialized: PelB > PelA > PelD > PelF > PelC. Interestingly, PelB was consistently the most efficient pectin lyase even at acidic conditions, despite having a higher pH optimum than PelA. In general, the highest extent of pectin degradation was observed for PelA and PelB. A systematic assessment of pH-temperature optima revealed significant differences in reaction optima among the different types of the pectin lyases, and homology modeling of the different versions of the pectin lyases supported that the slight sequence disparity amongst the copies, fosters structural differences in the active site region of the enzymes. Together with differences in thermal stability amongst the pectin lyases, these differences and the activity optimum variation easily supports the prevailing theory that this multigenecity is related to competitive fitness evolution providing the fungus with optimal tools for survival in different environments.

By providing a compendium of the degradation product profiles for the available *Aspergillus* pectin lyases on three different types of pectin (from apple, citrus, and sugar beet), which varied in degrees of methoxylation and acetylation, the present work added a new dimension to pectin lyase diversity characterization, and moreover provided a new angle to understand why fungi possess multiple copies of similar CAZymes. The discriminative LC-MS based substrate degradation pattern analysis provides support to the idea that the multigenecity is a prerequisite for the sophisticated biological “plasticity” that grants the fungus the ability to degrade an array of almost similar structural moieties, in this case pectin having different methoxylation and acetylation patterns. This interpretation agrees with the idea that the evolution of the CAZymes-secretome is an integral part of fungal speciation (Benoit et al., 2015; Barrett et al., 2020b). The product profiling method presented here may furthermore be useful as a novel tool in enzyme characterization as it enables discrimination between different enzymes with identical catalytic activity, and thus provides a new approach to continued exploration of the action of CAZymes on complex biomass substrates.

## Data Availability Statement

All datasets generated for this study are included in the article/Supplementary Material.

## Author Contributions

JH, AM, MS, and KK conceptualized and supervised the study. MS and KK expressed and purified the enzymes and determined their melting temperatures. TT performed experiments to determine substrate monosaccharide composition, enzyme pH-temperature optima, and kinetic constants. BZ determined initial rates on different pectin substrates and performed SDS-PAGE analysis. BZ, TT, and MS performed all *in silico* analyses. JH performed experiments and LC-MS and SEC analyses for product profiling and degradative pattern display. JH, AM, BZ, and TT analyzed all data. BZ, TT, and JH drafted the manuscript. AM, JH, MS, KK, and BZ revised the final manuscript. All authors contributed to the article and approved the submitted version.

## Conflict of Interest

MS and KK are employed by the company Novozymes A/S. The remaining authors declare that the research was conducted in the absence of any commercial or financial relationships that could be construed as a potential conflict of interest.

## Footnotes

1 https://cupp.info/cupp/PL1:12.1
