# Supplementary material for: Comparative Characterization of Aspergillus Pectin Lyases by Discriminative Substrate Degradation Profiling
Source: Front Bioeng Biotechnol. 2020 Jul 30;8:873. doi: 10.3389/fbioe.2020.00873 (PMC7406575; doi:10.3389/fbioe.2020.00873)
Supplement: Supplementary file 1 [file Table_1.DOCX]

Supplementary Material

Comparative characterization of *Aspergillus* pectin lyases by discriminative substrate degradation profiling

Birgitte Zeuner^1^, Thore Bach Thomsen^1^, Mary Ann Stringer^2^, Kristian B. R. M. Krogh^2^, Anne S. Meyer^1*^, Jesper Holck^1^

^1^Technical University of Denmark, Søltofts Plads 221, 2800 Kgs. Lyngby, Denmark

^2^ Novozymes A/S, Biologiens Vej 2, 2800 Kgs. Lyngby, Denmark

*** Correspondence:** asme@dtu.dk

**
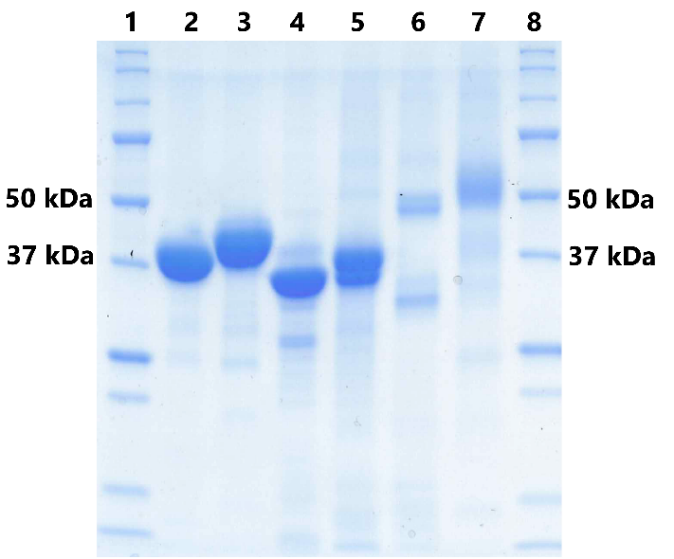
**

**Figure S1.** SDS-PAGE of the purified pectin lyases. Lane 1: marker; lane 2: *Aa*PelA; lane 3: *At*PelA; lane 4: *Al*PelB; lane 5: *Al*PelC; lane 6: *Al*PelD; lane 7: *Al*PelF; lane 8: marker.

|  | ***Aa*PelA** | ***At*PelA** | ***Al*PelB** | ***Al*PelC** | ***Al*PelD** | ***Al*PelF** |
| --- | --- | --- | --- | --- | --- | --- |
| ***Aa*PelA** | 100 | 75 | 68 | 49 | 66 | 48 |
| ***At*PelA** | 75 | 100 | 66 | 49 | 73 | 48 |
| ***Al*PelB** | 68 | 66 | 100 | 49 | 64 | 46 |
| ***Al*PelC** | 49 | 49 | 49 | 100 | 49 | 43 |
| ***Al*PelD** | 66 | 73 | 64 | 49 | 100 | 51 |
| ***Al*PelF** | 48 | 48 | 46 | 43 | 51 | 100 |

**Figure S2.** Pairwise sequence comparisons of the pectin lyases used in this work given as % identity.


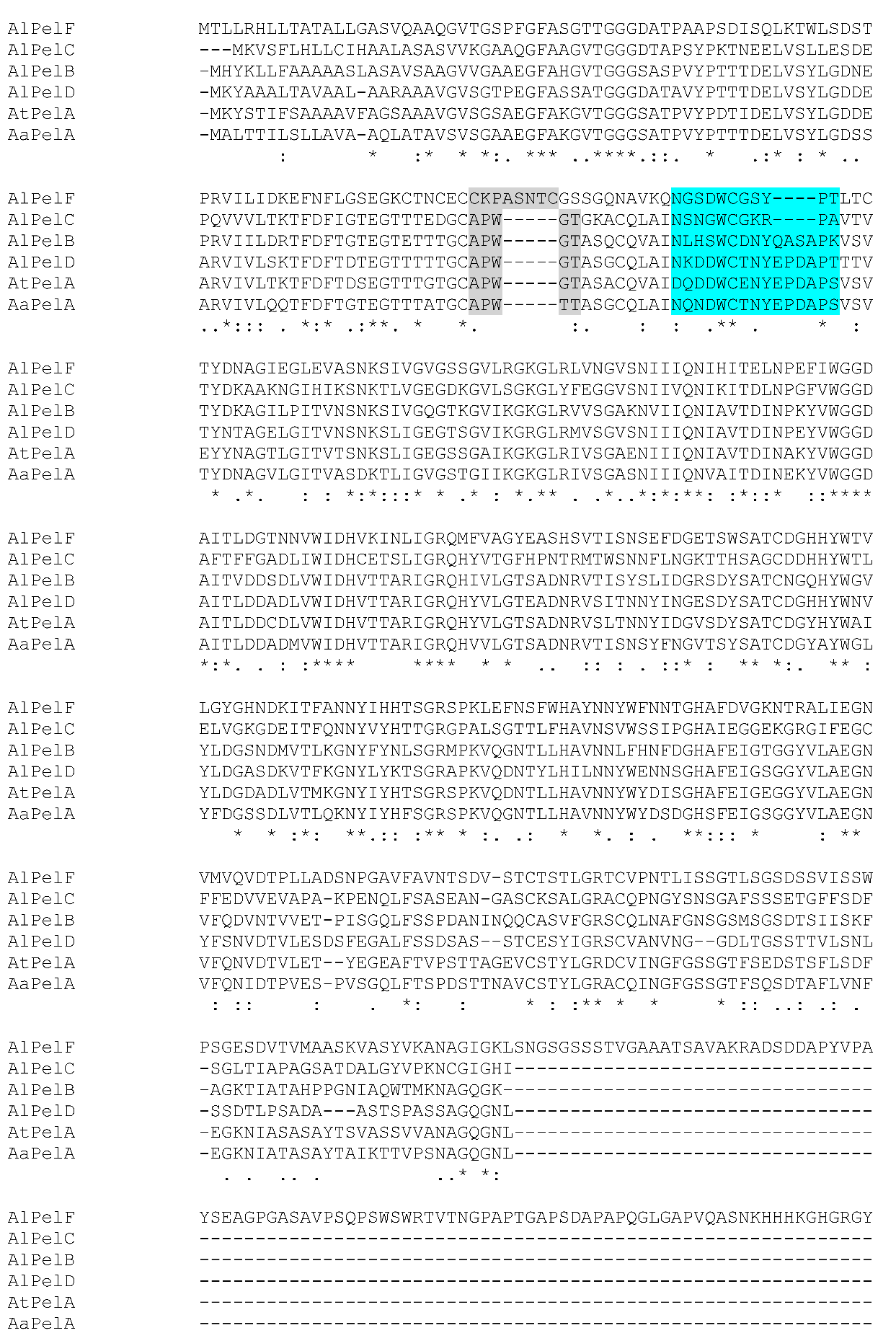


**Figure S3.** Multiple sequence alignment. The loop above the active site, where *Al*PelC and *Al*PelF are different from the other four pectin lyases when comparing the homology models (Figure 3), is indicated in blue. Another loop containing a conserved substrate-interacting Trp residue, where *Al*PelF is different from the other pectin lyases, is indicated in grey.

**
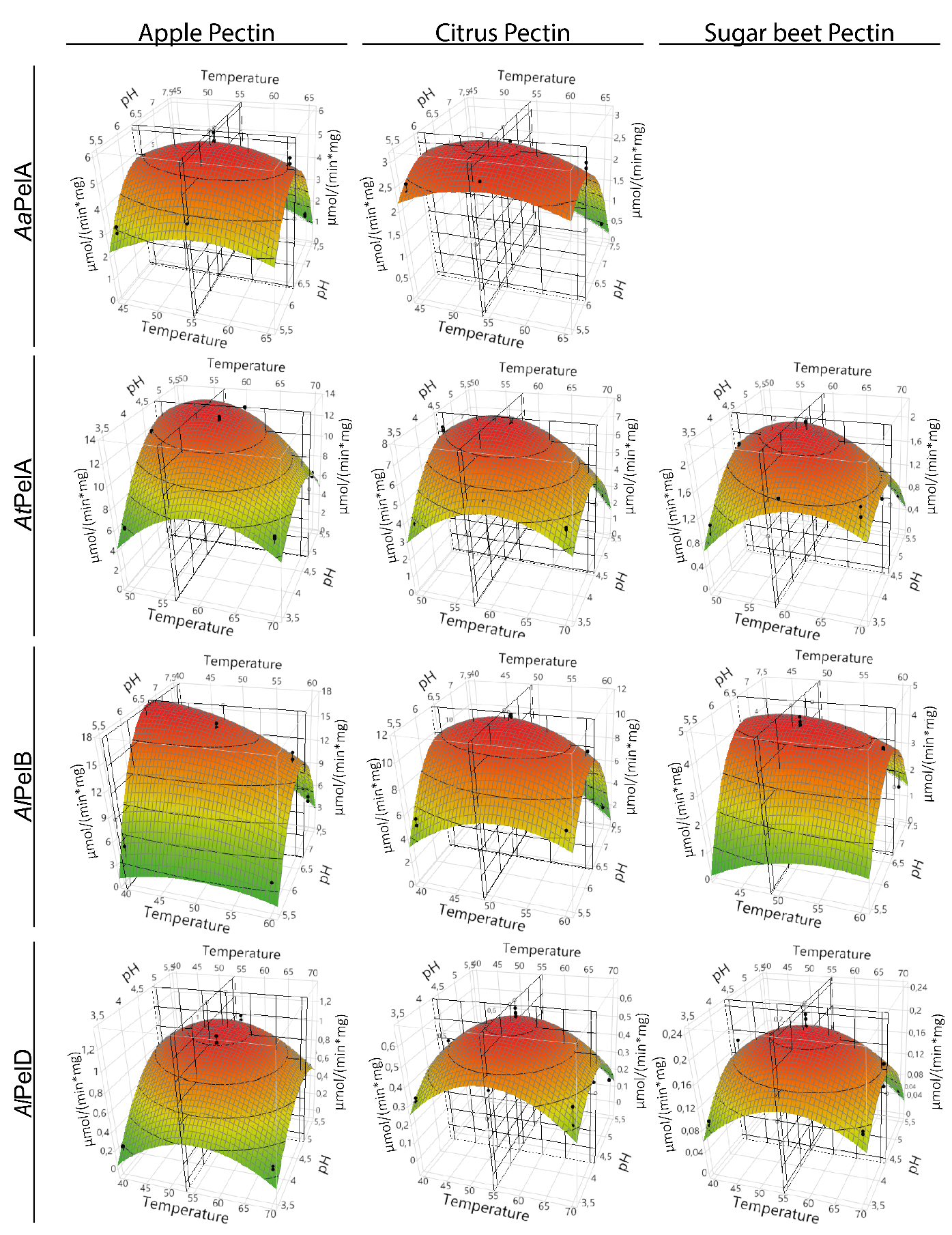
**

**Figure S4.** pH-temperature optima for *Aa*PelA, *At*PelA, *Al*PelB, and *Al*PelD on three different pectin substrates as estimated from the two-factor face-centered central composite design of experiment (CCF).

**
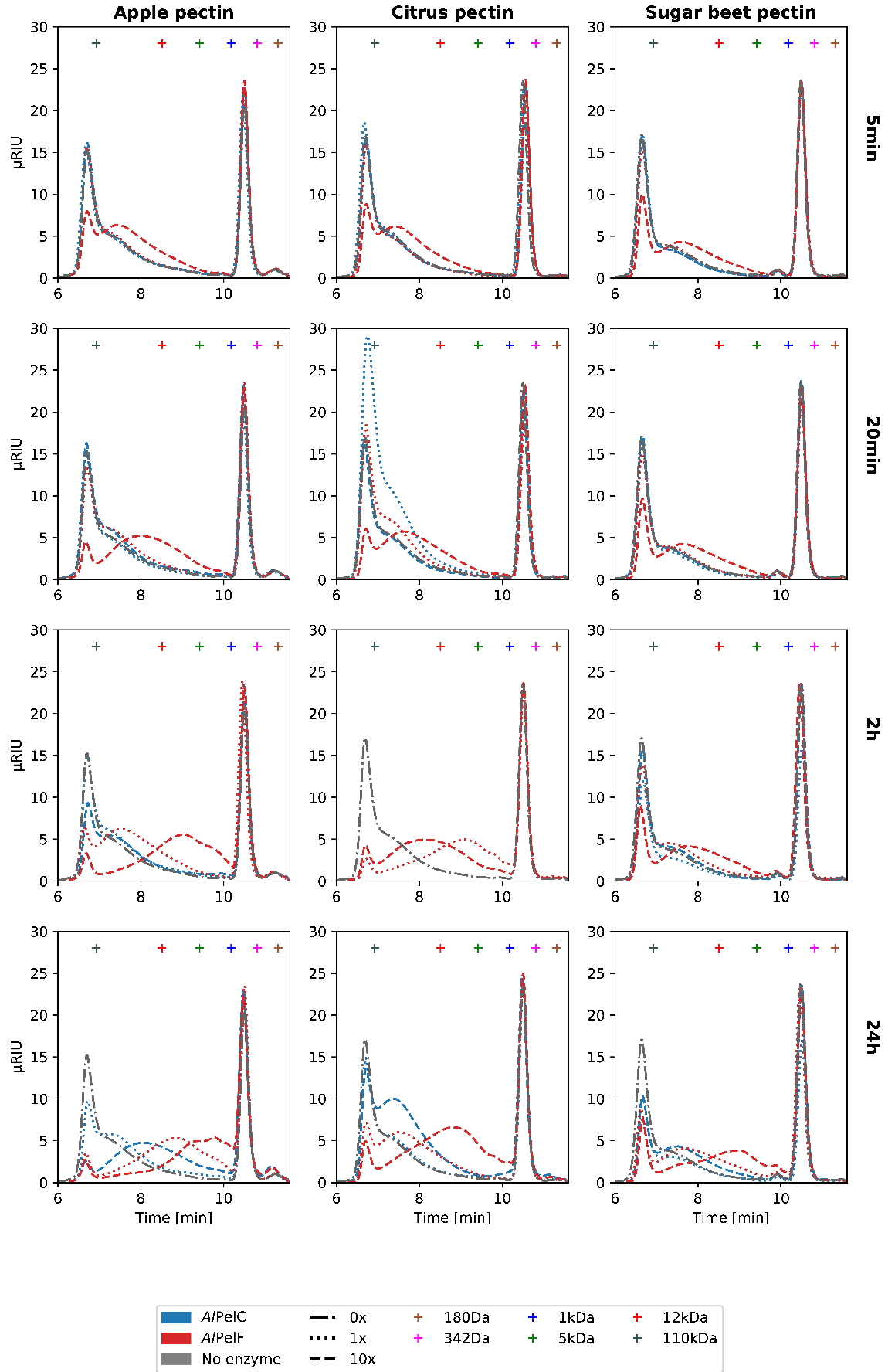
**

**Figure S5.** Size exclusion chromatograms comparing *Al*PelC (blue) and *Al*PelF (red) at 60 nM (short dashes) and 600 nM (long dashes) for degradation of 10 g/L apple pectin, citrus pectin, and sugar beet pectin. A negative control (‘No enzyme’) is shown in grey. Retention times of pullulan standard (180 Da to 110 kDa) are indicated by crosses.


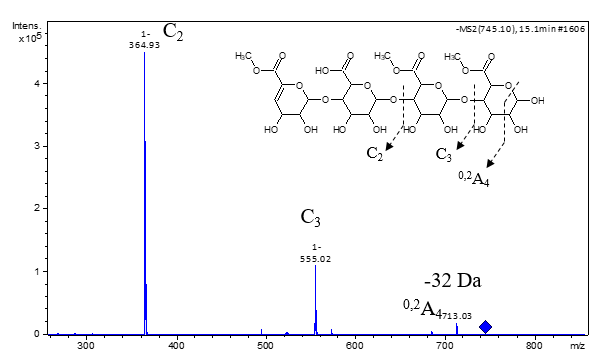


**Figure S6.** Negative ion mode ESI-MS^2^ of gA_4_m_3_.


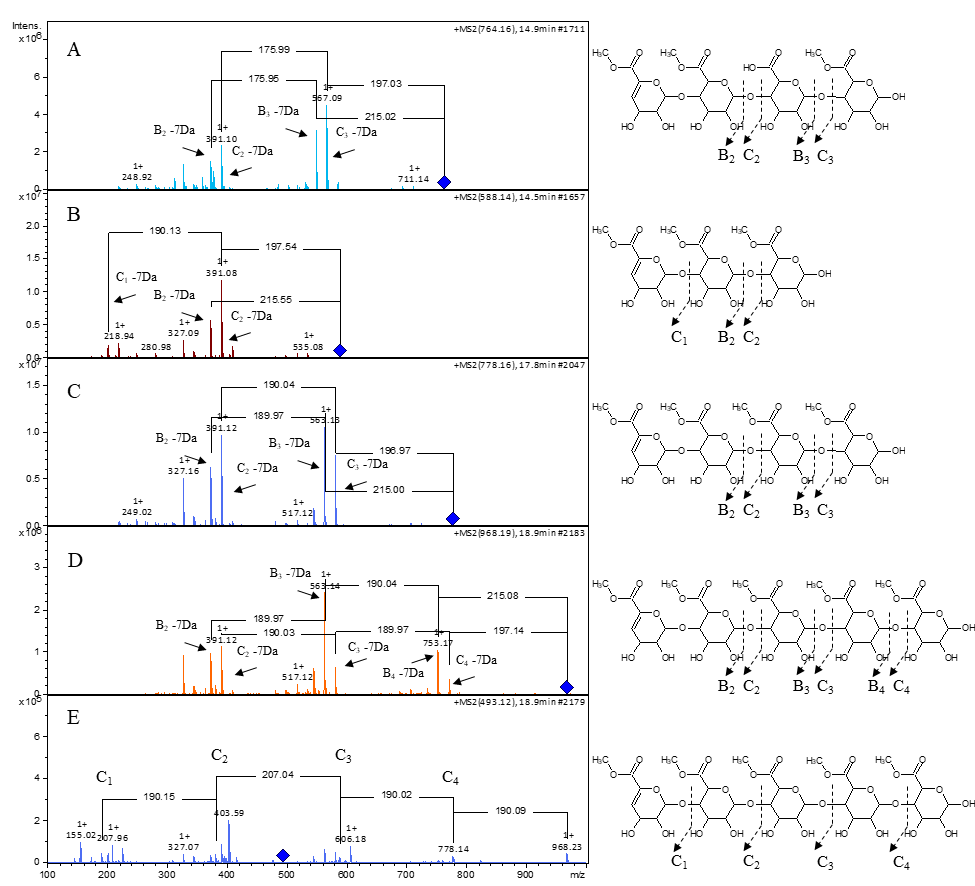


**Figure S7.** Positive ion mode ESI MS^2^ of ammonium adducts of A: gA_4_m_3_, B: gA_3_m_3_, C: gA_4_m_4_, D: gA_5_m_5_ (single charge), E: gA_5_m_5_ (double charge). Diamond indicates mother ion.


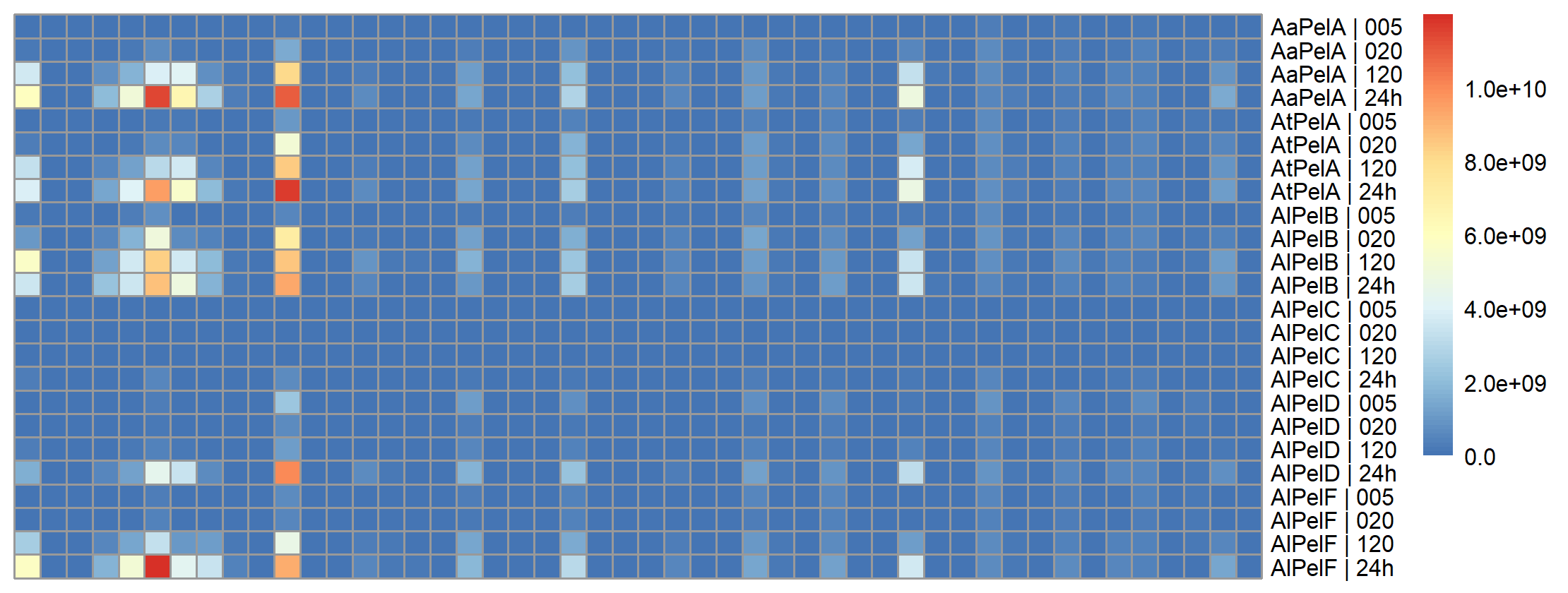


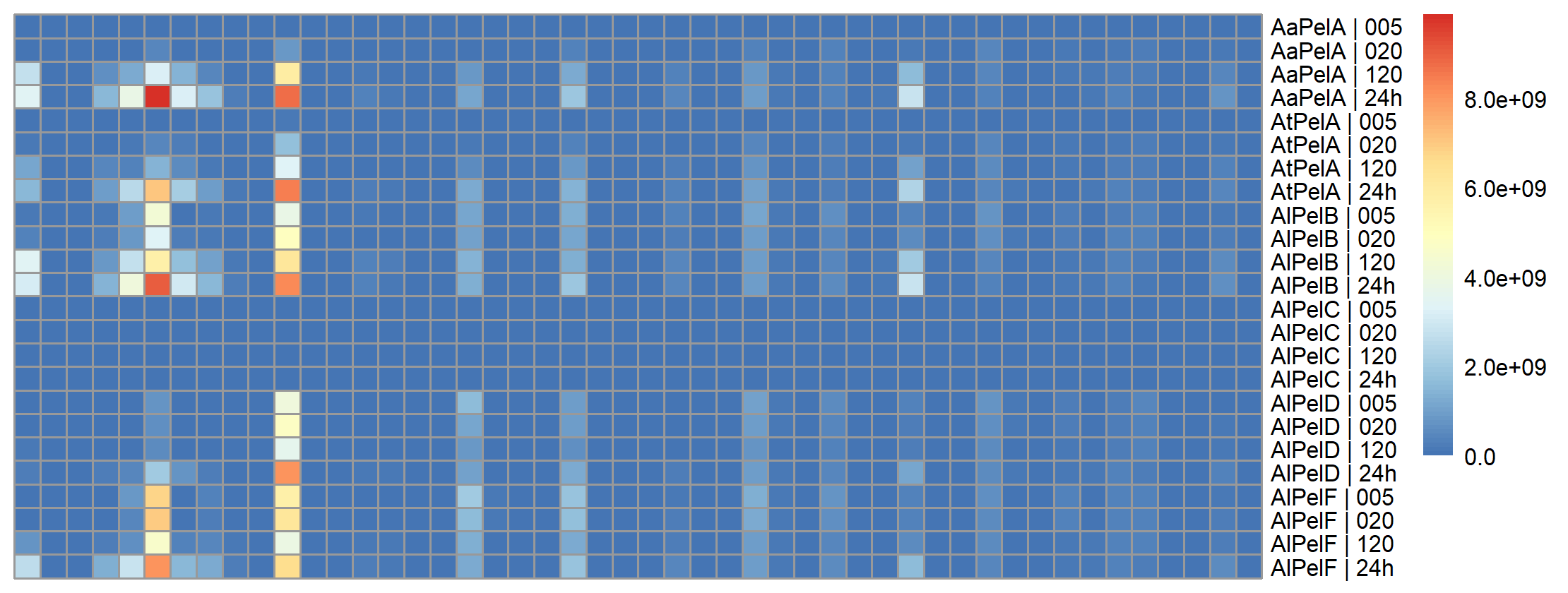


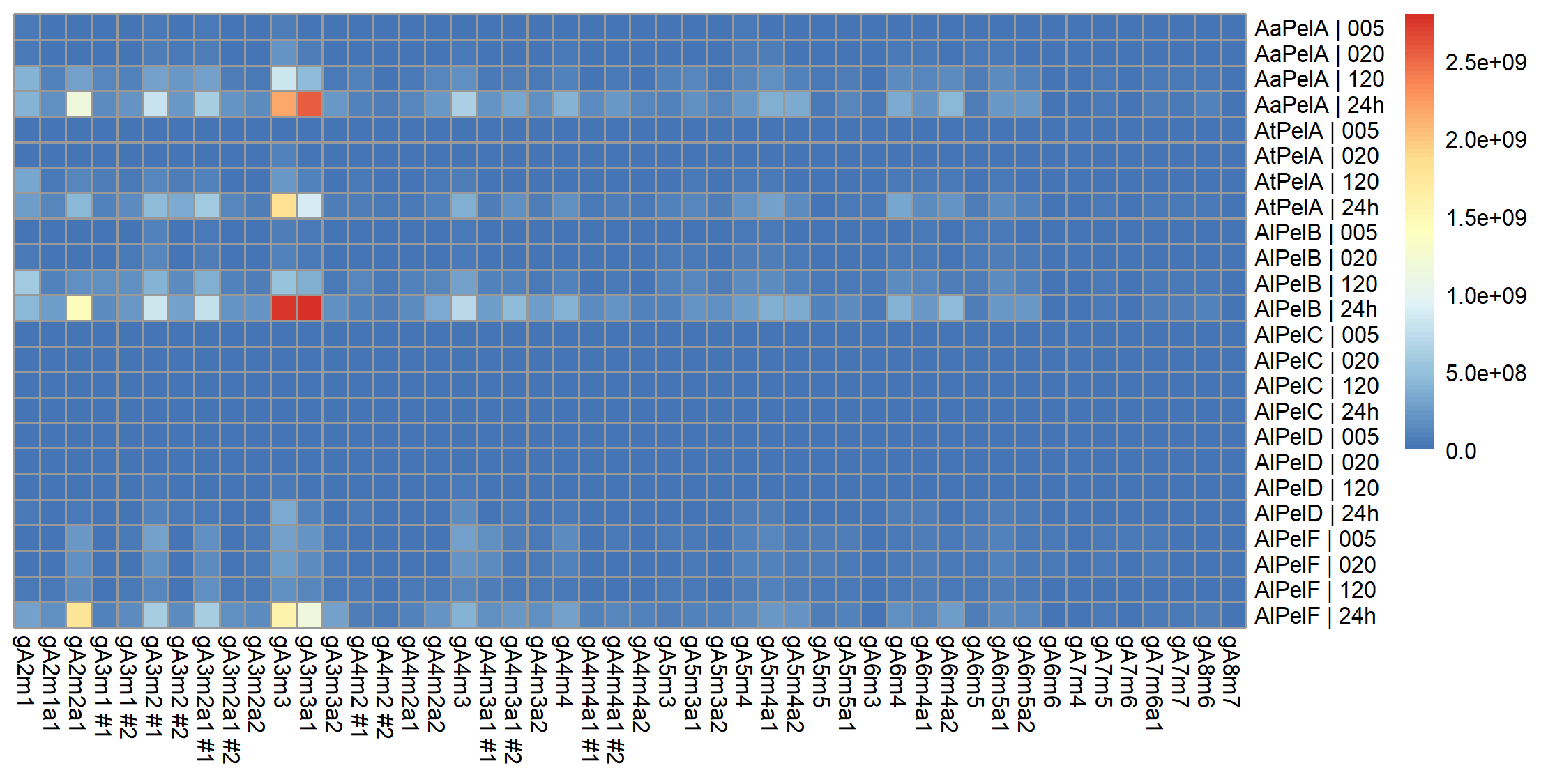


**Figure S8.** Observed peak intensities of identified compounds from positive ESI MS extracted ion chromatograms. Top: Apple pectin, middle: citrus pectin, bottom: sugar beet pectin.

**Table S1.** Optimal pH and temperature as well as kinetic constants determined for *Aspergillus* sp. pectin lyases in literature. Included here in uniform units for easy comparison to kinetic constants obtained in the current work. Substrate source and degree of methoxylation (DM) as well as reaction conditions used for determining kinetic constants are indicated. *K*_m_ is given in mM bonds and *k*_cat_ as µmol product released per µmol enzyme per second (s^-1^).

|  |  | **Optima** | | **Kinetic parameters and conditions used for kinetic analysis** | | | | | | |  |
| --- | --- | --- | --- | --- | --- | --- | --- | --- | --- | --- | --- |
| **Enzyme** | **Origin** | **pH_opt_** | ***T*_opt_** | **Substrate** | **DM** | **pH** | **T** | ***K*_m_** | ***k*_cat_** | ***k*_cat_/*K*_m_** | **Reference** |
|  |  |  | *°C* |  | *%* |  | *°C* | *mM* | *s^-1^* | *mM^-1^ s^-1^* |  |
| PelA | *Aspergillus niger* CBS 513.88 | 4.5 | 50 | - | - | - | - | - | - | - | (He et al., 2018) |
| PelA | *Aspergillus niger* CBS 120.49 | - | - | Pectin | 94.6 | 6.0 | 25 | 1.2 | 26 | 22 | (Kusters-van Someren et al., 1991) |
| PelA/PLII | Ultrazym® (*Aspergillus niger*) | 5.5-6.5 | - | Apple pectin | 94.2 | 6.0 | 25 | 1.5 | 27 | 18 | (van Houdenhoven, 1975; Kester and Visser, 1994) |
| PelB | *Aspergillus niger* CBS 120.49 | 8.5 | - | Pectin | 94.6 | 8.5 | 25 | 8.5 | 508 | 60 | (Kester and Visser, 1994) |
| PelD/PLI | Ultrazym® (*Aspergillus niger*) | 5.5-6.0 | - | Apple pectin | 94.2 | 6.0 | 25 | 10 | 78 | 8 | (van Houdenhoven, 1975; Gysler et al., 1990; Kester and Visser, 1994) |
| PelF | *Aspergillus niger* ZJ5 | 5.0 | 43 | Citrus pectin | - | 5.0 | 43 | 2.9^a^ | 26 | 9 | (Xu et al., 2015) |

^a^ Approximate value calculated from *K*_m_ given in mg/mL.

**References for Table S1:**

Gysler, C., Harmsen, J. A. M., Kester, H. C. M., Visser, J., and Heim, J. (1990). Isolation and structure of the pectin lyase D-encoding gene from Aspergillus niger. *Gene* 89, 101–108.

He, Y., Pan, L., and Wang, B. (2018). Efficient Over-expression and Application of High-performance Pectin Lyase by Screening Aspergillus niger Pectin Lyase Gene Family. *Biotechnol. Bioprocess Eng.* 23, 662–669. doi:10.1007/s12257-018-0387-1.

Kester, H. C. M., and Visser, J. (1994). Purification and characterization of pectin lyase B, a novel pectinolytic enzyme from Aspergillus niger. *FEMS Microbiol. Lett.* 120, 63–68. doi:10.1016/0378-1097(94)00176-6.

Kusters-van Someren, M. A., Harmsen, J. A. M., Kester, H. C. M., and Visser, J. (1991). Structure of the Aspergillus niger pelA gene and its expression in Aspergillus niger and Aspergillus nidulans. *Curr. Genet.* 20, 293–299. doi:10.1007/BF00318518.

van Houdenhoven, F. E. A. (1975). *Studies on pectin lyase. PhD thesis.* Agricultural University, Wageningen.

Xu, S. X., Qin, X., Liu, B., Zhang, D. Q., Zhang, W., Wu, K., et al. (2015). An acidic pectin lyase from Aspergillus niger with favourable efficiency in fruit juice clarification. *Lett. Appl. Microbiol.* 60, 181–187. doi:10.1111/lam.12357.

**Table S2.** Parameter estimates and model evaluation of the regression models generated by the two-factor face-centered central composite design of experiments (CCF) for the determination of combined pH-temperature optima for the pectin lyases on apple pectin (AP), citrus pectin (CP), or sugar beet pectin (SBP). The equation of the models is: activity [µmol/(min·mg)] = intercept + β_1_·*T* + β_2_·*T*^2^ + β_3_·pH + β_4_·pH^2^ + β_5_·pH·*T*, where the β-values are the parameters given below. The *R*^2^ indicates how well the model fits the data. The lack of fit is given as the *p-*value, where *p* < 0.05 indicates that the generated model has a significant lack of fit. An asterisk (*) indicates a statistically insignificant main factor (pH or *T*). Dash (-) indicates insignificant interactions, which were removed before remodeling the data. For *Aa*PelA on sugar beet pectin (SBP), no useful model could be made as the differences between the data points in the design were not statistically significant.

|  |  | **Parameter estimates** | | | | | | **Evaluation** | |
| --- | --- | --- | --- | --- | --- | --- | --- | --- | --- |
| **Enzyme** | **Substrate** | Intercept | *T* | *T*^2^ | pH | pH^2^ | *T*·pH | *R*^2^ | Lack of fit |
| ***Aa*PelA** | AP | 56 | -0.09* | -6.7 | -6.2 | -25 | -2.2 | 0.98 | 0.0084 |
|  | CP | 29 | -1.3 | -3.2 | -7.9 | -9.3 | -1.7 | 0.99 | 0.0073 |
|  | SBP | n.d. | n.d. | n.d. | n.d. | n.d. | n.d. | n.d. | n.d. |
| ***At*PelA** | AP | 135 | -12 | -28 | 15 | -29 | -17 | 0.98 | <0.0001 |
|  | CP | 78 | -6.3 | -15 | -1.3* | -22 | -8.8 | 0.97 | <0.0001 |
|  | SBP | 21 | -1.0 | -4.5 | -0.86* | -5.4 | -3.3 | 0.92 | 0.0009 |
| ***Al*PelB** | AP | 163 | -20 | -12 | 27 | -86 | -20 | 0.98 | <0.0001 |
|  | CP | 113 | -4.2 | -17 | -8.9 | -49 | -11 | 0.99 | 0.0055 |
|  | SBP | 44 | -1.1* | -5.1 | 7.2 | -20 | -5.5 | 0.98 | 0.1280 |
| ***Al*PelD** | AP | 10 | -0.40 | -2.9 | 2.3 | -2.8 | -0.70 | 0.98 | <0.0001 |
|  | CP | 6.1 | -0.17* | -1.8 | -0.60 | -1.8 | - | 0.94 | 0.0054 |
|  | SBP | 2.1 | -0.03* | -0.52 | -0.16 | -0.75 | -0.12 | 0.96 | 0.2646 |

**Table S3.** Amino acid sequence of *Aa*PelA.

>AaPelA

MALTTILSLLAVAAQLATAVSVSGAAEGFAKGVTGGGSATPVYPTTTDELVSYLGDSSARVIVLQQTFDFTGTEGTTTATGCAPWTTASGCQLAINQNDWCTNYEPDAPSVSVTYDNAGVLGITVASDKTLIGVGSTGIIKGKGLRIVSGASNIIIQNVAITDINEKYVWGGDAITLDDADMVWIDHVTTARIGRQHVVLGTSADNRVTISNSYFNGVTSYSATCDGYAYWGLYFDGSSDLVTLQKNYIYHFSGRSPKVQGNTLLHAVNNYWYDSDGHSFEIGSGGYVLAEGNVFQNIDTPVESPVSGQLFTSPDSTTNAVCSTYLGRACQINGFGSSGTFSQSDTAFLVNFEGKNIATASAYTAIKTTVPSNAGQGNL
